# Restoring NAD^+^ pathways in brain aging and neurodegenerative diseases: roles of vitamin B3 metabolites, gut microbiota, and exercise in cognitive health

**DOI:** 10.3389/fnut.2026.1865556

**Published:** 2026-09-23

**Authors:** Wu Zhang

**Affiliations:** College of Physical Education, Yan’an University, Yan’an, Shaanxi, China

**Keywords:** brain aging, exercise, gut microbiome dynamics, NAD^+^ repletion, neurodegenerative disease, vitamin B3 metabolites

## Abstract

Aging and neurodegenerative disorders (NDDs) are intricately linked to a gradual deterioration in nicotinamide adenine dinucleotide (NAD^+^) metabolism, compromised mitochondrial functionality, persistent inflammation, and modified gut–brain interactions. Derivatives of vitamin B3, such as niacin, nicotinamide, nicotinamide riboside, and nicotinamide mononucleotide, have been identified as pivotal NAD^+^ precursors that possess the potential to support cellular bioenergetic processes and modulate pathways associated with brain aging. Evidence from preclinical studies and emerging clinical investigations suggests that NAD^+^-enhancing interventions may improve mitochondrial function and modulate neuroinflammatory pathways, thereby supporting cellular processes involved in synaptic plasticity and cognitive function; however, their long-term efficacy and clinical relevance in NDDs remain to be fully established. Concurrently, physical exercise serves as a robust physiological stimulus that augments NAD^+^ biosynthesis, modulates hypoxia-inducible factor-1α-dependent adaptive responses, fosters neurotrophic signaling, and enhances both vascular and metabolic health of the brain. Furthermore, emerging evidence suggests that the gut microbiota directly contributes to NAD^+^ homeostasis by regulating vitamin B3 precursor availability, tryptophan metabolism, and host metabolic signaling, while also influencing the efficacy of NAD^+^-enhancing interventions, thereby affecting systemic inflammation, metabolic equilibrium, and the production of neuroactive metabolites. Collectively, the interaction among vitamin B3 supplementation, physical activity, and gut microbiota may represent a promising framework for investigating strategies aimed at supporting cognitive health and brain resilience during aging, although definitive therapeutic applications require further validation. This review integrates current molecular, preclinical, and clinical evidence to establish mechanistic links between NAD^+^ metabolism, brain aging, cognitive dysfunction, and neurodegeneration, while emphasizing current limitations and future translational challenges associated with combining vitamin B3 metabolites, exercise, and microbiome-targeted approaches.

## 1 Introduction

Cerebral senescence and neurodegenerative pathologies constitute one of the most urgent challenges in global health, with a rapidly escalating prevalence attributable to the aging demographic worldwide (1, 2). Conditions such as Alzheimer’s disease (AD), Parkinson’s disease (PD), and age-associated cognitive deterioration impose significant socioeconomic and healthcare strains, substantially diminishing both quality of life and autonomy among the elderly population (3, 4). Although considerable advances have been achieved in understanding the molecular pathology of these disorders, currently available therapeutic approaches remain largely symptomatic and have limited capacity to halt or reverse disease progression. Consequently, increasing attention has shifted toward identifying upstream biological mechanisms that drive brain aging and may serve as common therapeutic and preventive targets across multiple neurodegenerative disorders (NDDs) (5, 6). Among the earliest and most clinically meaningful features of brain aging is the slow deterioration of cognitive abilities, especially episodic memory, learning, processing speed, and executive function. These deficits mainly stem from progressive breakdown of neural circuits that are particularly susceptible, notably the entorhinal cortex–hippocampal network and the prefrontal cortex, both of which rely heavily on sustained mitochondrial ATP generation and effective metabolic homeostasis (7, 8). The entorhinal cortex serves as the principal cortical input to the hippocampus and is essential for memory encoding, consolidation, and spatial navigation, making this circuit one of the earliest and most vulnerable targets during both physiological brain aging and AD (9, 10). Progressive dysfunction within this network closely correlates with episodic memory decline and provides an important anatomical framework linking age-related metabolic impairment with cognitive deterioration. Additional findings also suggest that disturbance of neuronal energy metabolism occurs before visible neurodegeneration and plays a direct role in synaptic dysfunction, reduced neuronal communication, and ongoing cognitive decline. Consequently, defining mechanistic connections between metabolic impairment and cognitive dysfunction offers a critical physiological basis for explaining how age-associated changes in NAD^+^ metabolism may drive the progression of NDDs.

The aging process of the brain is marked by numerous interrelated biological characteristics, including progressive metabolic decline, mitochondrial dysfunction, persistent low-grade inflammation, compromised proteostasis, and diminished synaptic plasticity (11). Among these hallmarks, metabolic dysfunction has emerged as a central driver because neurons possess exceptionally high energetic demands and rely almost exclusively on mitochondrial oxidative phosphorylation to sustain synaptic transmission, axonal transport, and neuronal plasticity (12). Within this spectrum, metabolic dysregulation assumes a pivotal role, as neurons are highly energy-dependent entities that depend on the continuous production of mitochondrial ATP to maintain synaptic transmission and plasticity (13). Age-related declines in mitochondrial efficacy engender heightened oxidative stress, the accumulation of damaged macromolecules, and the activation of neuroinflammatory pathways, all of which collectively expedite cognitive decline (14, 15). These pathological events are further amplified by impaired DNA repair mechanisms and dysregulated cellular stress responses, ultimately establishing a self-perpetuating cycle of neuronal vulnerability. Importantly, accumulating evidence indicates that vulnerability to age-associated metabolic impairment is not consistent across either brain regions or neuronal cell types. Hippocampal and cortical neurons impose particularly high metabolic requirements and are thus especially susceptible to NAD^+^ depletion and mitochondrial dysfunction, while astrocytes, microglia, and oligodendrocytes follow distinct metabolic programs and antioxidant capabilities that shape disease progression in different ways (16, 17). Microglia, for instance, express relatively high levels of CD38, a principal NAD^+^-consuming enzyme, which helps connect chronic neuroinflammation with progressive NAD^+^ depletion. By contrast, astrocytes make substantial contributions to metabolic support, redox homeostasis, and neurotransmitter recycling, whereas oligodendrocytes depend on sufficient mitochondrial metabolism to maintain myelin integrity. Together, these cell-type-specific differences imply that restoring NAD^+^ metabolism could produce distinct therapeutic effects across different neural cell populations and across stages of neurodegeneration. Interestingly, tissue-specific responses to NAD^+^ depletion seem to vary markedly across excitable tissues. A recent study found that severe NAD^+^ depletion in skeletal muscle did not substantially disrupt muscle function or hasten organismal aging, implying that compensatory metabolic pathways may maintain skeletal muscle homeostasis even when NAD^+^ availability is reduced. By contrast, neurons appear substantially more susceptible to metabolic stress, since synaptic transmission depends on continuous ATP generation, tightly controlled Ca^2+^ signaling, and ongoing mitochondrial activity. One structural feature linked to this vulnerability is the dendritic spine: a highly dynamic postsynaptic compartment enriched with mitochondria and specialized endoplasmic reticulum, the spine apparatus, both of which are required for calcium homeostasis and synaptic plasticity. Aging and major NDDs reliably show dendritic spine loss, which closely tracks cognitive decline. Collectively, these findings indicate that maintaining synaptic integrity–rather than merely restoring tissue NAD^+^ levels–may be a key factor in effective neuroprotection (18–22).

An expanding body of evidence identifies declining NAD^+^ availability as a central integrative mechanism underlying brain aging (6, 16). NAD^+^ functions as an essential metabolic coenzyme participating in redox reactions, mitochondrial energy production, DNA repair, calcium signaling, and multiple intracellular signaling pathways regulated by sirtuins, poly(ADP-ribose) polymerases (PARPs), and CD38 (6, 23). With advancing age, intracellular NAD^+^ levels progressively decline because of reduced biosynthesis together with increased enzymatic consumption, leading to impaired mitochondrial bioenergetics, defective DNA repair, chronic inflammatory activation, and reduced cellular stress resistance (23, 24). Consequently, restoration of NAD^+^ homeostasis has emerged as a promising experimental approach for targeting multiple hallmarks of aging simultaneously, although its long-term efficacy and clinical applicability remain under active investigation. Mechanistically, restoration of NAD^+^ availability is thought to improve cellular resilience primarily through activation of downstream signaling pathways involving Sirtuin 1 (SIRT1), sirtuin 3 (SIRT3), PARPs, and AMP-activated protein kinase (AMPK), thereby promoting mitochondrial quality control, oxidative phosphorylation, mitophagy, genomic stability, and inflammatory resolution rather than exerting direct effects on neuronal plasticity itself (17, 25). These downstream mechanisms ultimately support neuronal survival, synaptic integrity, and cognitive function, although their relative contribution appears to differ among NDDs and remains incompletely understood. Investigations have demonstrated that restoration of NAD^+^ metabolism can attenuate cognitive impairment, reduce neuronal loss, and improve synaptic function in experimental models of AD; however, whether these findings translate into clinically meaningful benefits in humans remains unclear (5). These findings have stimulated growing interest in interventions capable of replenishing intracellular NAD^+^ pools, particularly vitamin B3-derived metabolites and related biosynthetic precursors. Derivatives of vitamin B3, including nicotinic acid (niacin), nicotinamide (NAM), nicotinamide riboside (NR), and nicotinamide mononucleotide (NMN), represent major NAD^+^ precursors that replenish intracellular NAD^+^ pools through distinct biosynthetic routes (26, 27). Although these molecules differ in absorption characteristics, pharmacokinetics, tissue distribution, and metabolic fate, they collectively contribute to maintaining NAD^+^ availability and supporting cellular energy metabolism (26).

However, their biological effects should not be interpreted as direct actions of NAD^+^ itself; rather, many neuroprotective effects are mediated through downstream NAD^+^-dependent enzymes and signaling networks, including sirtuins, PARPs, and mitochondrial quality-control pathways. Among available NAD^+^ precursors, NR and NMN have attracted substantial attention because of their ability to increase systemic NAD^+^ concentrations and their favorable safety profiles reported in early human studies (28). However, evidence regarding their effects on brain NAD^+^ availability and clinical outcomes in age-related cognitive decline remains insufficient. This distinction is particularly important because tissue-specific differences in NAD^+^ transport, precursor utilization, and NAD^+^-consuming enzyme activity may influence therapeutic outcomes. Conversely, niacin and NAM have longer histories of clinical use; however, their potential neuroprotective effects and optimal therapeutic applications remain under investigation (29). Beyond nutritional approaches, physical exercise has emerged as an important physiological regulator of NAD^+^ metabolism and brain resilience-associated pathways. Exercise activates energy-sensing pathways, including AMPK and sirtuin signaling, which are closely interconnected with NAD^+^ availability and mitochondrial adaptation (30, 31). Regular physical activity promotes mitochondrial biogenesis, improves cerebrovascular function, enhances neurotrophic signaling, and supports synaptic maintenance through pathways involving brain-derived neurotrophic factor (BDNF) and other adaptive responses. Moreover, exercise-induced metabolic stress regulates hypoxia-inducible factor-1α (HIF-1α), a transcriptional regulator involved in angiogenesis, mitochondrial adaptation, and neuronal resilience. Experimental evidence demonstrates that HIF-1α signaling is required for exercise-induced neuroprotection, highlighting its potential role as a mechanistic link between exercise adaptation and brain resilience (32). Even though exercise and NAD^+^ precursor supplementation target several shared molecular pathways, including mitochondrial function, regulation of oxidative stress, and control of inflammation, their combined impact is still a developing area of research. Accordingly, future studies should clarify whether exercise improves the effectiveness of interventions designed to increase NAD^+^, or whether particular exercise modalities, intensities, and durations are necessary to optimize NAD^+^-dependent adaptations. Age-related alterations in gut microbiota composition represent another important contributor to metabolic dysfunction and brain aging. Aging is associated with reduced microbial diversity, increased intestinal permeability, altered immune signaling, and changes in microbial metabolites that influence systemic inflammation and brain function (33).

Recent evidence indicates that the gut microbiota is not simply a downstream target of interventions related to NAD^+^, but may contribute to NAD^+^ homeostasis through several potential mechanisms. These include altering the availability of vitamin B3 precursors, modulating microbial tryptophan metabolism, generating NAD^+^-associated metabolites, and regulating host inflammatory and metabolic pathways. Consequently, the microbiota–NAD^+^ axis may function as a bidirectional communication network linking intestinal metabolism, systemic aging, and neuronal function. Moreover, both physical exercise and nutritional interventions can modify the composition of gut microorganisms and their metabolic activity, indicating possible synergistic interactions among lifestyle factors, microbial ecology, and NAD^+^ metabolism. Nonetheless, the evidence for direct causal links between changes in the microbiome and brain NAD^+^ availability is still scarce, underscoring a key translational gap that calls for longitudinal human studies. Taken together, the current body of evidence supports an integrated, though intricate, framework in which vitamin B3 metabolites, exercise, and the gut microbiota may interact to influence NAD^+^ availability, mitochondrial robustness, inflammatory control, and neuronal function. Even so, substantial uncertainties persist regarding the most effective dosing approaches, long-term safety, responses that vary by tissue, clinically relevant biomarkers, and the degree to which restoring NAD^+^ can avert cognitive decline rather than merely postpone it. This review aims to provide a comprehensive and critical evaluation of NAD^+^ decline as a central mechanism linking aging with cognitive impairment and NDDs. Specifically, we integrate molecular, cellular, preclinical, and clinical evidence to examine how vitamin B3-derived NAD^+^ precursors, exercise-based interventions, and gut microbiota modulation may collectively influence brain aging. Furthermore, we highlight disease-specific mechanisms, current knowledge gaps, and future translational challenges associated with NAD^+^-targeted preventive and therapeutic strategies for NDDs.

## 2 Literature search strategy and study selection

This manuscript was carried out as a comprehensive narrative review, with the goal of synthesizing current evidence on NAD^+^ metabolism, NAD^+^ precursors derived from vitamin B3, exercise interventions, interactions involving the gut microbiota, and their possible contributions to brain aging and NDDs. Although the review was not intended to function as a systematic review or meta-analysis, a structured search approach was implemented to locate pertinent experimental and clinical evidence and to enhance transparency about study selection. A thorough search of the scientific literature was conducted in the electronic databases PubMed/MEDLINE, Web of Science, Scopus, and Embase from their inception through July 2026. The search strategy used a combination of controlled vocabulary terms and free-text keywords related to NAD^+^ biology, aging, cognition, neurodegeneration, vitamin B3 metabolites, exercise, and the gut microbiota. The primary search terms combined: (“NAD^+^” OR “nicotinamide adenine dinucleotide” OR “NAD precursors” OR “nicotinamide riboside” OR “nicotinamide mononucleotide” OR “niacin” OR “nicotinamide”) AND (“brain aging” OR “cognitive decline” OR “neurodegeneration” OR “Alzheimer’s disease” OR “Parkinson’s disease” OR “neurodegenerative disorders”) AND (“exercise” OR “physical activity” OR “training”) AND (“gut microbiota” OR “microbiome” OR “gut-brain axis”). In addition, relevant publications were found through manual screening of the reference lists of selected articles and recent review papers. Studies were deemed eligible if they examined the association between NAD^+^ metabolism and neurological outcomes related to aging, including cognitive decline, neuronal dysfunction, neuroinflammation, mitochondrial impairment, synaptic changes, or NDDs mechanisms. Both preclinical research (cellular and animal models) and human studies were included to offer a translational perspective, with particular emphasis on studies assessing NAD^+^ precursors, exercise-related regulation of NAD^+^, and microbiota-driven metabolic pathways. Studies were excluded if they were not connected to neurological aging or neurodegenerative conditions, if they concentrated solely on non-neural tissues without mechanistic relevance to brain aging, if they lacked adequate methodological detail, or if they were published as conference abstracts, editorials, or non-peer-reviewed reports. Because this review sought to enable mechanistic integration rather than provide a quantitative synthesis, studies were chosen according to scientific relevance, mechanistic value, translational significance, and their capacity to reflect major advances or outstanding questions in the field. The available evidence was rigorously appraised and arranged into thematic sections covering: (1) biological mechanisms driving age-related declines in NAD^+^, (2) vitamin B3-based NAD^+^ restoration approaches, (3) regulation of NAD^+^ metabolism and neuroprotection through exercise, (4) interactions between the gut microbiota and NAD^+^, and (5) disease-specific implications across major NDDs. Particular emphasis was placed on separating findings obtained from cellular models, animal studies, and human clinical investigations, in order to prevent overextending conclusions about translational relevance.

## 3 NAD^+^ metabolism and its role in brain aging

NAD^+^ represents a pivotal metabolic cofactor that orchestrates redox reactions, maintains cellular energy equilibrium, and modulates signaling pathways essential for optimal brain functionality. Within the context of senescence, diminished NAD^+^ concentrations are profoundly correlated with mitochondrial dysfunction, oxidative stress, and compromised neuronal resilience. Furthermore, NAD^+^ acts as a substrate for enzymes including sirtuins, PARPs, and CD38, which play crucial roles in DNA repair, epigenetic modulation, and calcium signaling. The perturbation of NAD^+^ metabolism is implicated in the pathophysiological mechanisms underlying NDDs, such as those manifested in AD and PD. Preclinical evidence suggests that restoring NAD^+^ availability may influence several processes relevant to brain aging, including mitochondrial function, inflammatory signaling, and cellular stress responses; however, the extent to which these effects translate into meaningful clinical benefits remains uncertain (6, 16, 17). Importantly, NAD^+^ should be viewed mainly as a metabolic regulator and a signaling substrate, rather than as a direct effector of neuronal plasticity or cognitive processes. Numerous reported benefits of NAD^+^ restoration including enhanced mitochondrial function, support of synaptic maintenance, and increased neuronal resilience, are driven by NAD^+^-dependent enzymes such as sirtuins, PARPs, and CD38, which govern downstream pathways related to mitochondrial quality control, genomic stability, inflammatory regulation, and cellular stress adaptation (34, 35). A decline in NAD^+^ with age may trigger a cascade of linked pathological processes, such as diminished mitochondrial ATP production, greater accumulation of reactive oxygen species (ROS), decreased DNA repair capacity, and sustained inflammatory activation. These changes can then impair neuronal function, compromise synaptic integrity, and reduce cognitive performance, thereby offering a mechanistic connection between NAD^+^ depletion, brain aging, and susceptibility to neurodegeneration (6, 12). However, disturbances in NAD^+^ balance are unlikely to affect all brain regions and cell populations in a uniform manner.

Neurons, owing to their exceptionally high energetic requirements and their dependence on mitochondrial oxidative metabolism, may be especially vulnerable to age-related decreases in NAD^+^. By contrast, glial cells show distinct metabolic properties and NAD^+^-associated enzyme expression patterns. For instance, microglia express high levels of CD38, a principal NAD^+^-consuming enzyme linked to inflammatory activation, whereas astrocytes and oligodendrocytes support metabolism, sustain neuronal function, and help maintain myelin homeostasis (12, 17, 36, 37). In addition, the extent to which NAD^+^-dependent enzymes contribute may differ depending on the cellular context. Sirtuins, particularly SIRT1 and SIRT3, participate in mitochondrial control, antioxidant defenses, and stress adaptation, while PARP activation supports DNA repair but can drive NAD^+^ depletion when DNA damage is excessive. Similarly, CD38-mediated NAD^+^ consumption has been increasingly linked to age-related inflammatory changes and metabolic dysfunction (34, 36). Accordingly, NAD^+^ metabolism has attracted interest as a potential intervention target because modulation of this pathway could influence multiple interconnected hallmarks of aging. Targeting NAD^+^ metabolism is particularly compelling as it presents a comprehensive systems-level strategy rather than concentrating solely on isolated downstream pathological characteristics. However, this rationale is based largely on mechanistic and preclinical findings, and it should not be interpreted as evidence that NAD^+^-targeted interventions can currently prevent or treat neurodegenerative disease in humans. Augmenting NAD^+^ availability may enhance mitochondrial biogenesis, support oxidative phosphorylation, and modulate sirtuin-dependent and inflammatory pathways in experimental settings (16, 17). Nevertheless, these beneficial effects should be interpreted as indirect consequences of NAD^+^-dependent signaling activation, including regulation of mitochondrial quality control, inflammatory pathways, and neuronal stress responses, rather than direct actions of NAD^+^ on neuronal structures (25, 35). Investigations have substantiated that repletion of NAD^+^ can alleviate cognitive impairments, diminish neuronal loss, and enhance synaptic functionality in models of AD (16). Whether comparable effects occur in patients with AD or other NDDs remains to be established. These revelations have stimulated the advancement of interventions aimed at boosting NAD^+^ levels, particularly through metabolites of vitamin B3 and associated precursors. Derivatives of vitamin B3, such as nicotinic acid (niacin), NAM, NR, and NMN, function as pivotal precursors for NAD^+^ through distinct biosynthetic pathways (17, 18). Although these compounds exhibit variation in terms of bioavailability, pharmacokinetics, and tissue distribution, they collectively play a significant role in the replenishment of intracellular NAD^+^ levels (17). Among the various compounds, NR and NMN have garnered considerable scholarly interest due to their efficacy in augmenting systemic levels of NAD^+^ and their commendable safety profiles observed in preliminary clinical investigations (19). Nevertheless, increases in systemic NAD^+^-related metabolites do not necessarily demonstrate equivalent restoration of NAD^+^ within specific brain regions, and clinical evidence for neurological efficacy remains limited. Concurrently, niacin and NAM have been historically employed in clinical settings, although their extensive neuroprotective implications remain subjects of ongoing research (20).

### 3.1 NAD^+^ biosynthesis pathways

NAD^+^ is synthesized through three principal biosynthetic pathways: the *de novo* pathway originating from tryptophan, the Preiss–Handler pathway derived from nicotinic acid, and the salvage pathway that utilizes NAM and its associated precursors. The *de novo* pathway encompasses several enzymatic reactions that convert tryptophan into quinolinic acid and ultimately yield NAD^+^; however, it exhibits relative inefficiency within the central nervous system. The Preiss–Handler pathway employs dietary sources of nicotinic acid, whereas the salvage pathway, orchestrated by nicotinamide phosphoribosyltransferase (NAMPT), represents the primary source of NAD^+^ in mammalian tissues. NR and NMN serve as important NAD^+^ precursors that enter salvage-related pathways and can increase intracellular NAD^+^ availability, although their efficiency depends on tissue-specific transport, enzymatic activity, and metabolic context. An age-related decline in NAMPT enzymatic activity results in diminished NAD^+^ synthesis, thereby potentially contributing to metabolic dysfunction. Interventions targeting these pathways have increased NAD^+^-related metabolites in preclinical models and early clinical studies, although whether such changes consistently translate into improved cognitive outcomes remains uncertain (26, 35, 38).

### 3.2 Age-related NAD^+^ decline mechanisms

Aging is correlated with a gradual reduction in NAD^+^ concentrations attributable to various converging mechanisms. The augmented activity of NAD^+^-depleting enzymes such as CD38 and PARPs markedly diminishes intracellular NAD^+^ reservoirs. Chronic DNA damage associated with aging results in sustained PARP activation, which further accelerates the consumption of NAD^+^. Moreover, the diminished expression and functionality of NAMPT hinder the salvage pathway, thereby restricting NAD^+^ biosynthesis. Mitochondrial dysfunction coupled with oxidative stress intensifies this decline by increasing metabolic demands and impairing cellular repair mechanisms. Inflammation, recognized as a characteristic of aging (inflammaging), also enhances the expression of CD38 in immune and neural cells. However, NAD^+^ depletion does not occur uniformly across all brain regions or cell types. Neurons, due to their high energetic demands, may be particularly vulnerable to impaired NAD^+^ availability, whereas microglia, astrocytes, and oligodendrocytes display distinct NAD^+^ metabolic profiles that may influence their contribution to aging-related neuronal dysfunction (17, 36). Collectively, these processes disrupt cellular energy homeostasis, augment neuronal susceptibility, and contribute to cognitive deterioration. Consequently, interventions aimed at mitigating NAD^+^ depletion mechanisms represent a mechanistically plausible avenue for investigation in the context of age-related NDDs (16, 17).

### 3.3 Role of NAD^+^ in neuronal energy metabolism

NAD^+^ assumes a crucial function in neuronal energy metabolism by serving as a fundamental cofactor in the processes of glycolysis, the tricarboxylic acid (TCA) cycle, and oxidative phosphorylation. Neurons, characterized by their high energy dependency, necessitate efficient ATP synthesis to uphold ion gradients, facilitate neurotransmission, and promote synaptic activity. NAD^+^ enables the transfer of electrons in redox reactions, thereby bolstering mitochondrial ATP production. A decrease in NAD^+^ concentrations disrupts mitochondrial respiration, resulting in diminished ATP synthesis and an escalation in the generation of ROS. This metabolic impairment is implicated in neuronal injury and cognitive decline. Moreover, NAD^+^ availability influences mitochondrial biogenesis and quality control through downstream activation of NAD^+^-dependent sirtuin signaling, particularly SIRT1 and SIRT3, thereby enhancing cellular resilience. The restoration of NAD^+^ concentrations has been demonstrated to augment mitochondrial functionality, mitigate oxidative stress, and promote neuronal viability in the contexts of aging and NDDs (6, 16, 39).

### 3.4 Sirtuins, PARPs, and CD38 in brain aging

Sirtuins, PARPs, and CD38 represent significant enzymes that consume NAD^+^ and play a pivotal role in the regulation of cerebral aging. Sirtuins (SIRT1–7) function as NAD^+^-dependent deacetylases, influencing gene expression, mitochondrial functionality, and cellular resilience to stressors (40). Notably, SIRT1 and SIRT3 are particularly instrumental in enhancing neuroprotection and maintaining metabolic equilibrium (41). Conversely, PARPs are activated as a consequence of DNA lesions and utilize substantial quantities of NAD^+^ during the reparative processes, thereby exacerbating NAD^+^ depletion in conditions of chronic stress (42). CD38, a prominent NAD^+^ hydrolase, exhibits increased expression with advancing age and inflammatory responses, thereby significantly diminishing the availability of NAD^+^. Among these pathways, age-associated CD38 upregulation and reduced NAMPT activity represent relatively consistent mechanisms contributing to NAD^+^ decline, whereas therapeutic modulation of individual NAD^+^-consuming enzymes remains an emerging area requiring further validation in human studies (17, 36). The disequilibrium between NAD^+^ consumption and biosynthesis culminates in compromised cellular functions and NDDs. The strategic targeting of these enzymes through the activation of sirtuins or the inhibition of PARPs and CD38, has therefore been proposed as a potential strategy for modifying NAD^+^ metabolism, although its therapeutic relevance to human brain aging remains to be established (17, 43).

### 3.5 Impact on synaptic function and neuroplasticity

NAD^+^ metabolism contributes to the maintenance of synaptic functionality and neuroplasticity through regulation of mitochondrial energy metabolism, oxidative stress responses, and downstream signaling pathways involved in neuronal adaptation (44). Sufficient concentrations of NAD^+^ are essential for the sustenance of mitochondrial vitality and the production of ATP, thereby facilitating effective synaptic transmission and the manifestation of plastic alterations (45). Activation of NAD^+^-dependent sirtuins, particularly SIRT1, may influence synaptic architecture, dendritic spine maintenance, and neurotrophic signaling pathways, including BDNF-related mechanisms (46). In contrast, the depletion of NAD^+^ adversely affects synaptic signaling, diminishes dendritic complexity, and disrupts inter-neuronal communication (47). The heightened activity of CD38 and PARPs further intensifies synaptic impairment through mechanisms involving calcium dysregulation and energy depletion. The augmentation of NAD^+^ levels has shown effects on synaptic and cognitive phenotypes predominantly in experimental models, supporting further investigation of NAD^+^ metabolism as a potential target in brain aging and NDDs (35). Thus, the beneficial effects of NAD^+^ restoration on cognition are likely mediated indirectly through improved mitochondrial function, reduced cellular stress, and enhanced neuronal signaling rather than through direct regulation of synaptic structures by NAD^+^ itself (Figure 1).

**FIGURE 1:**
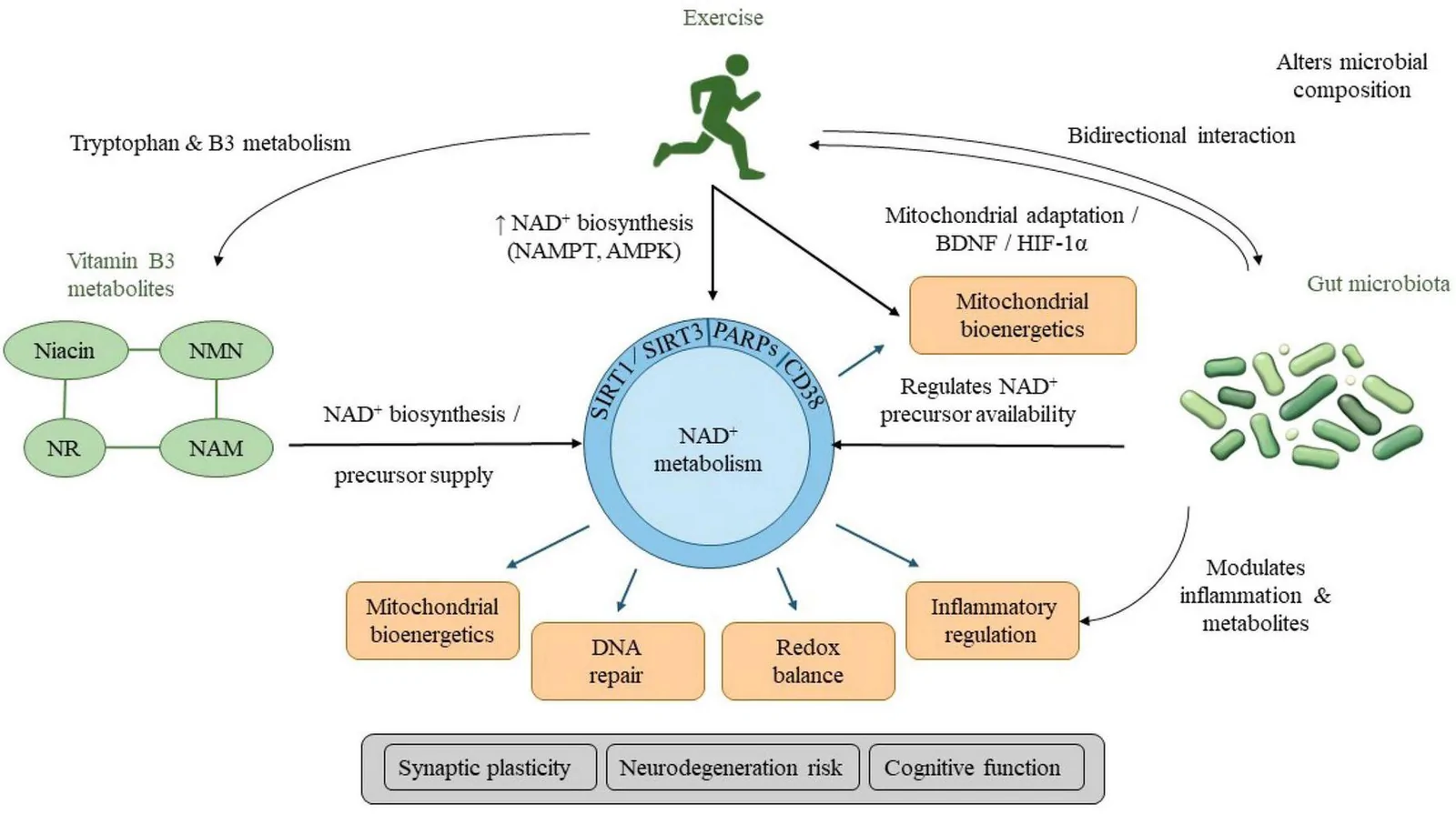
Integrated NAD^+^-centered network linking vitamin B3 metabolites, exercise, gut microbiota, and brain aging. Vitamin B3–derived precursors, including niacin, nicotinamide, nicotinamide riboside, and nicotinamide mononucleotide, together with exercise and gut microbiota, converge to regulate NAD^+^ availability. NAD^+^ acts as a central metabolic hub that modulates downstream pathways through NAD^+^-dependent enzymes, including sirtuins, PARPs, and CD38. These pathways collectively influence mitochondrial function, redox balance, DNA repair, and inflammatory responses, ultimately affecting synaptic plasticity, cognitive function, and vulnerability to neurodegeneration. Bidirectional interactions among exercise and gut microbiota, as well as microbiota-driven regulation of precursor availability, are highlighted to emphasize system-level integration. NAD^+^, nicotinamide adenine dinucleotide; NAM, nicotinamide; NR, nicotinamide riboside; NMN, nicotinamide mononucleotide; SIRT, sirtuin; PARP, poly(ADP-ribose) polymerase; AMPK, AMP-activated protein kinase; BDNF, brain-derived neurotrophic factor; HIF-1α, hypoxia-inducible factor 1-alpha; ROS, reactive oxygen species.

## 4 Vitamin B3 metabolites as NAD^+^ precursors

### 4.1 Nicotinic acid (niacin)

Nicotinic acid, which is commonly referred to as niacin, represents one of the earliest recognized forms of vitamin B3 and serves as a well-established precursor for NAD^+^ through the Preiss–Handler metabolic pathway. Following its absorption, nicotinic acid undergoes conversion to nicotinic acid mononucleotide and subsequently to NAD^+^. Clinically, niacin has been extensively utilized due to its lipid-lowering properties, especially in its ability to diminish LDL cholesterol levels while elevating HDL levels. Concerning the phenomenon of brain aging, niacin contributes to NAD^+^ replenishment and may influence cellular energy metabolism and DNA repair capacity through NAD^+^-dependent pathways. Nevertheless, its application is frequently constrained by adverse effects, such as flushing, which is mediated by the release of prostaglandins. In spite of these limitations, niacin supplementation could influence pathways relevant to vascular and cellular stress responses, but direct neuroprotective effects and preventive benefits in NDDs remain insufficiently established (26, 48).

### 4.2 NAM

NAM serves as a crucial precursor for NAD^+^, being produced as a byproduct of enzymatic reactions that consume NAD^+^ and subsequently recycled via the salvage pathway. The conversion of NAM to NMN is facilitated by the rate-limiting enzyme NAMPT, which ultimately culminates in the synthesis of NAD^+^. The accessibility, cost-effectiveness, and reported ability of NAM to reach the central nervous system make it an attractive candidate for applications within the field of neurology. Empirical evidence indicates that NAM can confer neuroprotective effects by diminishing oxidative stress, inhibiting apoptotic pathways, and enhancing mitochondrial functionality. Nevertheless, elevated concentrations of NAM may lead to the suppression of sirtuin activity due to feedback inhibition, which could potentially restrict its long-term therapeutic effectiveness. Therefore, achieving an optimal balance in NAM supplementation to enhance NAD^+^ production while avoiding the inhibition of beneficial signaling pathways is a critical consideration in clinical contexts (17, 38).

### 4.3 NR

NR constitutes a naturally occurring precursor of NAD^+^, present in minimal concentrations in dietary sources such as milk. It undergoes conversion to NMN through the catalytic action of nicotinamide riboside kinases and is subsequently transformed into NAD^+^ via the salvage metabolic pathway. The compound NR has garnered substantial scholarly attention due to its elevated bioavailability and its capacity to effectively augment NAD^+^ concentrations across various tissues, including cerebral regions. Most evidence supporting these effects is derived from preclinical models, whereas human studies have primarily demonstrated increases in NAD^+^ metabolites and acceptable short-term safety profiles rather than definitive neuroprotective outcomes. In models of neurodegeneration, NR has been reported to improve neuronal survival markers, cognitive performance, and synaptic function in experimental models of neurodegeneration; however, translation of these findings to humans remains uncertain (6, 27).

### 4.4 NMN

NMN serves as a direct intermediate for NAD^+^ and functions as a highly effective precursor within the salvage pathway. The synthesis of NMN occurs through the enzymatic action of NAMPT on NAM and is subsequently converted into NAD^+^ by NMN adenylyltransferases with remarkable efficiency. NMN supplementation has been shown to increase NAD^+^ availability in several tissues, particularly in experimental models, although evidence regarding direct effects within human brain tissue remains limited. In experimental animal models, NMN administration has been observed to enhance insulin sensitivity, mitigate oxidative stress, and improve cognitive performance. Furthermore, NMN has been associated with changes in neuronal energy metabolism and inflammatory responses in experimental studies, but the extent to which these findings translate into clinically meaningful neurological benefits remains unclear. Overall, vitamin B3-derived NAD^+^ precursors differ substantially in their metabolic routes, bioavailability, and translational evidence. While NR and NMN have attracted considerable interest because of their capacity to increase NAD^+^ metabolites, most neuroprotective evidence remains derived from cellular and animal studies. Niacin and NAM have longer clinical histories but present distinct pharmacological limitations. Therefore, the therapeutic potential of vitamin B3 metabolites should be interpreted according to precursor-specific characteristics, tissue accessibility, disease context, and the level of clinical validation. Current clinical investigations are evaluating their safety, pharmacokinetics, effects on NAD^+^-related biomarkers, and potential functional outcomes, but definitive evidence for disease-modifying effects in NDDs is not yet available (17, 35).

## 5 Exercise as a modulator of NAD^+^ and brain health

### 5.1 Exercise-induced NAD^+^ biosynthesis

Exercise has been reported to influence NAD^+^ homeostasis, partly through modulation of the salvage pathway and NAMPT expression, although the magnitude of these effects appears to depend on exercise modality, tissue type, age, and metabolic status (49). Both acute and chronic exercise can modify NAD^+^ metabolism in peripheral tissues, particularly skeletal muscle; however, whether comparable increases occur within human brain tissue remains insufficiently established (50). Importantly, recent evidence suggests that changes in skeletal muscle NAD^+^ availability do not necessarily translate into accelerated muscle aging or functional decline, highlighting the need to avoid assuming identical NAD^+^ requirements across different tissues, particularly between muscle and brain. Exercise-associated changes in NAD^+^ availability may support mitochondrial oxidative capacity and cellular adaptation to metabolic stress, although the extent of these effects is tissue- and context-dependent. The elevation of NAD^+^ induced by exercise is also correlated with increased sirtuin activity, which fosters mitochondrial biogenesis and bolsters antioxidant defenses. Exercise-induced changes in oxygen availability also activate HIF-1α, a key regulator of metabolic adaptation and neuroplasticity. HIF-1α signaling interacts with NAD^+^-dependent pathways and may contribute to exercise-induced neuroprotection by regulating angiogenesis, mitochondrial adaptation, and neuronal resilience (32, 51, 52). Moreover, physical activity contributes positively to systemic metabolic health, indirectly facilitating NAD^+^ homeostasis by mitigating inflammation and oxidative injury (53, 54). Collectively, these mechanisms provide a plausible biological basis for the relationship between exercise, cellular energy metabolism, and brain resilience during aging. However, the extent to which exercise-induced changes in NAD^+^ directly contribute to cognitive or disease-related outcomes in humans remains uncertain.

### 5.2 Neurotrophic factors

Engagement in physical exercise considerably amplifies the expression of neurotrophic factors, notably BDNF and NGF, both of which are critical for neuronal survival, differentiation, and synaptic plasticity (55). BDNF is pivotal in facilitating learning and memory processes by fostering long-term potentiation (LTP) and the formation of dendritic spines. Regular physical activity elevates BDNF concentrations in vital brain regions, such as the hippocampus, thus enhancing cognitive function (56). In a parallel manner, NGF is instrumental in the survival and maintenance of cholinergic neurons, which are particularly susceptible to degeneration in conditions such as AD. The upregulation of these neurotrophic factors is, in part, a result of exercise-induced activation of metabolic sensors, including AMPK and NAD^+^-dependent SIRT1 signaling, which may contribute to the regulation of neurotrophic pathways such as BDNF (57). These molecular adaptations are proposed to facilitate neurogenesis and enhance neuronal resilience, although the contribution of individual pathways varies according to exercise intensity, duration, and disease context. Importantly, evidence linking these molecular adaptations to clinically meaningful changes in neurodegenerative disease progression remains limited.

### 5.3 Cognitive benefits in aging and dementia models

Extensive evidence from animal models and human clinical studies indicates that physical exercise can improve cognitive performance and preserve brain function during aging, although the magnitude of these effects varies according to exercise modality, intensity, duration, and disease stage (58). Regular physical activity is associated with improved memory, executive function, and processing speed, and may reduce vulnerability to age-related cognitive decline and NDDs such as AD and PD; however, definitive disease-preventive effects remain to be fully established in clinical populations (59). Experimental studies provide mechanistic evidence that exercise can promote hippocampal neurogenesis, influence amyloid-β pathology, and attenuate neuroinflammatory responses, although these findings cannot be assumed to translate directly to clinical disease modification (60). In contrast, human clinical studies have demonstrated more heterogeneous effects, with aerobic and resistance exercise generally improving cognitive outcomes and functional capacity, although effects on disease progression remain influenced by differences in intervention protocols and participant characteristics (61). These cognitive benefits may be mediated by multiple interconnected mechanisms, including exercise-induced modulation of NAD^+^ metabolism, improved mitochondrial bioenergetics, enhanced neurotrophic signaling, and regulation of inflammatory pathways. However, the relative contribution of NAD^+^ restoration to exercise-induced cognitive improvement remains incompletely defined, particularly in humans. Furthermore, physical exercise contributes to vascular adaptation and cerebral perfusion, which may support neuronal resilience and cognitive maintenance during aging.

Overall, exercise should be considered a multifactorial regulator of brain aging rather than solely an NAD^+^-enhancing intervention. Its beneficial effects likely arise from the coordinated interaction of NAD^+^ metabolism, mitochondrial adaptation, neurotrophic signaling, vascular regulation, and inflammatory control. Importantly, peripheral changes in NAD^+^ metabolism, including those observed in skeletal muscle, should not be assumed to directly reflect identical alterations within the brain, highlighting the need for tissue-specific investigations to clarify the translational relevance of exercise-induced NAD^+^ modulation.

## 6 Effects of vitamin B3 (NAM and NR) supplementation on aging-related outcomes

### 6.1 Evidence from preclinical and clinical studies

Current evidence regarding vitamin B3-derived NAD^+^ precursors, particularly NR and NAM, indicates strong mechanistic support from preclinical models and increasing but still preliminary evidence from human studies. Experimental investigations consistently demonstrate that NAD^+^ restoration can improve mitochondrial function, reduce inflammatory signaling, and enhance cellular resilience during aging-related stress. However, translation into clinically meaningful outcomes, particularly cognitive improvement and disease modification, remains incomplete. Therefore, evidence from animal models and human trials should be interpreted according to biological context, tissue specificity, intervention duration, and the distinction between molecular biomarkers and functional outcomes. In aged mice exposed to chronic stress, NR supplementation partially improved immune resilience by increasing B- and T-cell populations and reducing stress-associated hematological alterations. However, NR also increased anxiety-like behaviors without producing measurable improvements in cognitive performance, suggesting that NAD^+^ restoration may exert context-dependent effects influenced by environmental stressors and physiological status (62) (Table 1). Peripheral artery disease, which is associated with vascular dysfunction, endothelial impairment, and increased risk of cognitive vulnerability in older adults, has also been investigated as a clinical model for evaluating NAD^+^ augmentation strategies. In a human intervention study, short-term NR supplementation improved markers of endothelial function, cerebrovascular responsiveness, and selected cognitive outcomes. Mechanistic analyzes suggested reduced oxidative stress, enhanced nitric oxide availability, and improved mitochondrial efficiency within endothelial cells (63). However, the clinical relevance of these vascular and metabolic improvements requires confirmation in larger and longer-duration trials.

**TABLE 1:** Effects of vitamin B3 (nicotinamide and nicotinamide riboside) supplementation on aging-related outcomes: mechanistic insights, translational evidence, and unresolved questions from preclinical and clinical studies.

Population/ model	Intervention	Key mechanisms	Main findings	Evidence synthesis and remaining uncertainties	References
Aged mice under chronic physiological stress	6 weeks nicotinamide riboside (NR) supplementation	↑ NAD^+^ levels; immune and hematological modulation	↑ B/T cells, ↓ thrombocytopenia, ↑ anxiety-like behavior (stress sensitivity), ↔ cognitive function	Preclinical evidence suggests that NR may enhance immune resilience while producing context-dependent effects on stress responses. The interaction between NAD^+^ supplementation and physiological stress conditions requires further investigation.	(62)
Older adults with peripheral artery disease (PAD) (4-week trial, *n* = 8)	Oral NR supplementation	↑ NAD^+^ →↑ endothelial function, ↓ oxidative stress, ↑ nitric oxide, ↑ mitochondrial function	↑ endothelial function, ↑ cerebrovascular response, ↑ cognitive performance, ↓ oxidative stress	Early clinical evidence supports potential vascular and cognitive benefits; however, conclusions remain limited by small sample size, open-label design, and lack of long-term controlled trials.	(63)
Qprt-deficient mice (impaired NAD^+^ synthesis model)	NR supplementation	↑ NAD^+^ restoration; improved mitochondrial function; enhanced meiosis and DNA repair	↑ NAD^+^ levels, ↓ germ cell apoptosis, ↑ spermatogenesis, ↑ DNA repair and meiotic progression	Mechanistic preclinical studies strongly support NAD^+^ replenishment as a strategy for preserving reproductive aging phenotypes, although translation to human aging remains unexplored.	(64)
Aged female mice (20–22 months) with tibial fracture surgery	Nicotinamide (NAM) pre- and post-treatment	↑ NAD^+^; ↓ neuroinflammation (IL-1β, CD38); ↑ SIRT1; ↓ oxidative stress; ↑ BDNF; improved synaptic plasticity	↑ LTP, ↑ dendritic spine density, ↑ cognitive function, ↓ neuroinflammation, ↓ oxidative stress	Evidence indicates that NAD^+^ precursors may counteract inflammation-associated cognitive decline; however, clinical relevance for postoperative delirium remains to be established.	(65)
Mouse oocytes (*in vitro* aging model)	NR supplementation (200 μM, 24 h)	↑ NAD^+^/SIRT1 pathway; ↓ ROS; ↑ mitochondrial function; improved spindle assembly and DNA integrity	↓ ROS, ↑ mitochondrial function, ↓ DNA damage (γH2AX), ↑ oocyte quality, ↑ embryonic development potential	Preclinical reproductive studies consistently identify oxidative stress reduction as a major mechanism of protection, but human clinical evidence is currently lacking.	(66)
Healthy older adults (randomized crossover trial, 6 weeks)	Oral NR (500 mg, 2 × /day)	↑ NAD^+^ in neuronal extracellular vesicles; modulation of insulin-Akt signaling; ↓ neuroinflammatory kinases (pJNK, pERK1/2)	↑ NAD^+^ levels (neuronal), ↓ Aβ42, ↓ pJNK, ↓ pERK1/2, ↓ neurodegenerative biomarkers	Human evidence demonstrates successful NAD^+^ target engagement in neuronal biomarkers; however, whether biomarker improvements translate into functional neurological benefits remains uncertain.	(67)
Middle-aged mice + mouse/human muscle cells	NR supplementation (4 weeks)	↑ NAD^+^; ↑ mitochondrial function; ↑ cellular energetics; improved myogenesis	↑ muscle NAD^+^, ↑ endurance, ↑ activity, ↑ grip strength, ↑ mitochondrial respiration, ↑ myogenic differentiation	Preclinical evidence supports potential applications for sarcopenia and muscle aging, but optimal dosing, duration, and clinical effectiveness require confirmation.	(68)
Aged mice (intestinal stem cell model)	NR supplementation (6 weeks)	↑ NAD^+^/SIRT1/mTORC1 axis; enhanced stem cell function and regeneration	↑ intestinal stem cell proliferation, ↑ regeneration capacity, ↑ SIRT1/mTORC1 activity, ↓ age-related decline	Mechanistic evidence highlights the NAD^+^–SIRT1–mTORC1 pathway as a key mediator of tissue rejuvenation, although tissue-specific responses remain incompletely understood.	(69)
Healthy middle-aged and older adults (randomized, double-blind crossover trial)	NR supplementation (2 × 6 weeks)	↑ NAD^+^ bioavailability; modulation of metabolic and cardiovascular pathways	↑ NAD^+^ levels, ↔ tolerability (well-tolerated), ↓ blood pressure (trend), ↓ arterial stiffness (trend)	Clinical trials consistently demonstrate safety and NAD^+^ elevation, but evidence for meaningful improvements in healthspan-related outcomes remains preliminary.	(71)
Older adults with subjective cognitive decline and mild cognitive impairment (8-week RCT)	Oral NR (1 g/day)	↑ NAD^+^; modulation of neurodegenerative biomarkers (pTau217); limited cognitive pathway effects	↓ pTau217, ↔ cognition, ↔ GFAP/NfL, ↑ physical activity (step count)	Human studies suggest selective effects on Alzheimer’s disease-related biomarkers without consistent cognitive benefits, indicating a gap between molecular effects and clinical outcomes.	(72)
Older adults with MCI (10-week randomized placebo-controlled trial)	NR dose escalation to 1 g/day	↑ NAD^+^; effects on cerebral blood flow; epigenetic modulation (DNA methylation, aging clocks)	↑ NAD^+^ levels, ↔ cognition, ↓ cerebral blood flow (DMN), ↑ DNA methylation, ↓ epigenetic age	Current clinical evidence supports safety and pharmacological efficacy for NAD^+^ enhancement, but larger and longer-duration trials are needed to determine cognitive and functional benefits.	(70)

NR, nicotinamide riboside; NAM, nicotinamide; NAD^+^, nicotinamide adenine dinucleotide; PAD, peripheral artery disease; Qprt, quinolinate phosphoribosyl transferase; LTP, long-term potentiation; ROS, reactive oxygen species; BDNF, brain-derived neurotrophic factor; NEVs, neuronal extracellular vesicles; Aβ42, amyloid beta 42; pJNK, phosphorylated c-Jun N-terminal kinase; pERK1/2, phosphorylated extracellular signal-regulated kinases 1/2; SIRT1, sirtuin 1; mTORC1, mechanistic target of rapamycin complex 1; GFAP, glial fibrillary acidic protein; NfL, neurofilament light chain; pTau217, phosphorylated tau 217; RBANS, repeatable battery for the assessment of neuropsychological status; MoCA, montreal cognitive assessment; CBF, cerebral blood flow; DMN, default mode network. Across preclinical and clinical studies, NAD^+^ precursor supplementation consistently enhances NAD^+^ availability and modulates interconnected pathways involving mitochondrial function, oxidative stress regulation, inflammation, and cellular resilience. However, translational outcomes remain heterogeneous, with stronger evidence for biochemical target engagement than for improvements in functional aging-related outcomes in humans. Differences in age, disease status, tissue specificity, intervention duration, and environmental stressors may contribute to variability across studies.

Studies targeting NAD^+^ biosynthetic pathways have further demonstrated the importance of NAD^+^ availability for maintaining tissue homeostasis during aging. In Qprt-deficient mice, disruption of *de novo* NAD^+^ synthesis resulted in age-dependent reproductive abnormalities associated with mitochondrial dysfunction, impaired DNA repair, and increased apoptosis. Importantly, NR supplementation restored NAD^+^ levels and partially rescued these defects (64). Although this model is not directly related to brain aging, it provides evidence that NAD^+^ availability represents a fundamental determinant of cellular resilience across aging tissues. In an aged mouse model of postoperative delirium, NAM administration attenuated surgery-induced cognitive impairment by reducing neuroinflammation, restoring hippocampal synaptic plasticity, increasing BDNF-related signaling, and modulating CD38/SIRT1-associated pathways (65). These findings provide mechanistic evidence that NAD^+^-related pathways may influence acute cognitive vulnerability in aging; however, their relevance to chronic human neurodegenerative disease remains uncertain.

Postovulatory aging of oocytes provides additional evidence that NAD^+^ depletion contributes to age-associated cellular dysfunction outside the nervous system. In an *in vitro* mouse model, NR supplementation restored NAD^+^ levels, reduced oxidative stress, improved mitochondrial function, and preserved spindle and chromosomal integrity through activation of NAD^+^/SIRT1-related signaling pathways (66). Although these findings support the broader role of NAD^+^ restoration in cellular aging, their direct relevance to neurodegenerative processes remains indirect and requires cautious interpretation. Human biomarker studies have provided preliminary evidence that NR can increase NAD^+^ availability in neuronal-derived extracellular vesicles and modify molecular signatures associated with neurodegenerative risk. In healthy older adults, oral NR supplementation increased neuronal extracellular vesicle NAD^+^ concentrations and reduced selected markers related to neuroinflammation and insulin resistance, including amyloid-β 42, phosphorylated c-Jun N-terminal kinase, and phosphorylated extracellular signal-regulated kinase 1/2 (67). These findings suggest that NR may influence brain-related metabolic pathways; however, extracellular vesicle biomarkers should be interpreted as indirect indicators of central nervous system responses rather than direct measurements of neuronal NAD^+^ restoration. Because skeletal muscle is frequently used as a surrogate tissue for evaluating systemic NAD^+^ metabolism, it is important to distinguish peripheral metabolic effects from brain-specific outcomes. Short-term NR supplementation in middle-aged mice and human muscle stem cells increased muscle NAD^+^ availability, enhanced mitochondrial respiration, improved endurance-related parameters, and promoted myogenic capacity (68). However, recent evidence indicates that reduced skeletal muscle NAD^+^ availability does not necessarily accelerate muscle aging or impair muscle function, emphasizing that NAD^+^ requirements and biological consequences may differ substantially between tissues. Therefore, findings from skeletal muscle should not be directly extrapolated to neuronal aging, where metabolic demands, mitochondrial organization, and vulnerability to oxidative stress are distinct.

Beyond muscle and brain, NR supplementation has also been investigated in other aging-related tissues. In aged mice, chronic NR administration restored intestinal stem cell function, improved proliferative capacity, and enhanced tissue regeneration following injury through mechanisms involving the NAD^+^/SIRT1/mechanistic target of rapamycin complex 1 pathway (69). These findings support the concept that NAD^+^ availability contributes to tissue maintenance during aging; however, the extent to which intestinal NAD^+^ regulation influences brain aging through gut–brain interactions remains an important area for future investigation. Clinical trials in humans have consistently demonstrated that NR supplementation is safe, well tolerated, and capable of increasing systemic NAD^+^ availability (70–72). Nevertheless, these biomarker and safety findings should not be interpreted as evidence of established neuroprotective or disease-modifying efficacy. Indeed, restoration of NAD^+^ biomarkers has not consistently translated into measurable improvements in cognition or disease-related outcomes.

In a randomized crossover trial involving middle-aged and older adults, chronic NR administration enhanced NAD^+^ metabolism and suggested potential vascular benefits, including improvements in arterial stiffness and blood pressure parameters, although larger studies are required to establish clinical efficacy (71). In older adults with subjective cognitive decline or mild cognitive impairment (MCI), NR supplementation increased NAD^+^ availability and modified selected molecular biomarkers, including reductions in plasma phosphorylated tau 217 (pTau217). However, short-term supplementation did not significantly improve global cognitive performance or most neurodegenerative biomarkers, highlighting the current gap between metabolic engagement and functional neurological outcomes (72). Similarly, in a pilot study of older individuals with MCI, NR effectively increased blood NAD^+^ concentrations but failed to produce significant improvements in cognitive scores or physical performance measures, despite exploratory changes in epigenetic aging markers (70).

Collectively, current evidence indicates that vitamin B3-derived NAD^+^ precursors reliably enhance NAD^+^ availability and influence mitochondrial, inflammatory, and metabolic pathways across multiple tissues. However, evidence for clinically meaningful cognitive improvement remains preliminary. The discrepancy between robust mechanistic effects observed in experimental models and limited functional benefits in human trials emphasizes the need for longer interventions, optimized dosing strategies, biomarker-guided approaches, and careful consideration of tissue-specific NAD^+^ biology. Accordingly, NR and NAM should currently be regarded as investigational approaches for modifying NAD^+^-related aging biology rather than established therapies for cognitive decline or neurodegenerative disease.

### 6.2 Critical appraisal of current evidence on vitamin B3-derived NAD^+^ precursors supplementation in aging

Although vitamin B3-derived NAD^+^ precursors have demonstrated consistent biological activity in restoring NAD^+^ availability, improving mitochondrial-related pathways, and modulating inflammatory signaling, the current evidence does not yet establish their efficacy as disease-modifying interventions for age-related cognitive decline or NDDs. A critical distinction should be made between molecular target engagement and clinically meaningful functional outcomes. Most preclinical studies demonstrate robust effects on NAD^+^ metabolism, mitochondrial function, oxidative stress, and inflammatory pathways; however, translation of these findings into improvements in cognition or neurological outcomes in humans remains limited (71–73). Human studies to date have primarily focused on safety, pharmacokinetics, and biomarker responses rather than definitive clinical endpoints. Randomized controlled trials have generally shown that NR and NMN supplementation can safely increase circulating NAD^+^ metabolites; however, improvements in cognitive performance, neurodegenerative progression, or functional independence have not been consistently demonstrated (71, 73, 74). This discrepancy may reflect several factors, including short intervention periods (typically weeks to a few months), relatively small sample sizes, heterogeneous participant characteristics, and the use of surrogate biomarkers rather than direct measures of brain function (71, 72, 75). Several limitations currently restrict interpretation of NAD^+^ precursor studies. First, optimal dosing strategies remain uncertain, as different precursors (niacin, NAM, NR, and NMN) exhibit distinct pharmacokinetic profiles, tissue distribution patterns, and metabolic fates (27, 35, 76). Whether higher NAD^+^ restoration necessarily produces greater neuroprotective effects remains unresolved, particularly because excessive NAD^+^ precursor availability may influence feedback regulation of NAD^+^-consuming enzymes such as sirtuins and PARPs. Second, tissue specificity represents a major consideration. Increases in peripheral NAD^+^ concentrations do not necessarily indicate equivalent restoration within the brain, where the blood–brain barrier, cellular metabolism, and region-specific vulnerability may influence biological responses (77, 78). Therefore, future studies should prioritize brain-relevant biomarkers, including neuronal extracellular vesicles, neuroimaging-based metabolic measures, and validated indicators of synaptic and inflammatory changes. Third, the biological context of NAD^+^ decline should be considered. Gradual NAD^+^ reduction during physiological aging may differ substantially from acute NAD^+^ depletion caused by severe DNA damage, inflammation, or experimental manipulation. Consequently, interventions effective in accelerated aging models may not produce identical outcomes in normal human aging.

Another important challenge is the limited understanding of long-term safety and preventive potential. While short-term studies indicate favorable tolerability, prolonged NAD^+^ enhancement over years remains insufficiently investigated, particularly regarding metabolic adaptation, cancer-related pathways, and age-dependent differences in response (35, 75). Future longitudinal studies should determine whether NAD^+^ restoration can delay cognitive decline before clinical symptoms emerge or whether its benefits are primarily restricted to individuals with established metabolic dysfunction. This distinction is particularly important because evidence of target engagement does not necessarily indicate that an intervention will alter the trajectory of cognitive aging or neurodegenerative disease. Finally, methodological differences among studies, including variations in precursor type, dose, intervention duration, participant age, baseline NAD^+^ status, and outcome measures, currently limit direct comparisons. Integration of NAD^+^ precursor supplementation with complementary interventions, particularly exercise and microbiome-targeted approaches, may represent a more physiologically relevant strategy because NAD^+^ metabolism is regulated by interconnected metabolic networks rather than isolated pathways (79, 80). Overall, current evidence supports NAD^+^ precursor supplementation as a promising metabolic intervention capable of modifying aging-associated biological processes; however, its translation into effective prevention or treatment strategies for cognitive decline and NDDs requires confirmation through larger, longer-term, and mechanistically guided clinical trials.

### 6.3 Molecular mechanisms of NAD^+^ repletion via NR in aging

The biological effects of NR supplementation are primarily mediated by restoration of intracellular NAD^+^ availability, which subsequently influences multiple NAD^+^-dependent signaling networks involved in cellular maintenance. However, these mechanisms should not be interpreted as direct actions of NAD^+^ itself; rather, NAD^+^ functions mainly by regulating downstream enzymes, including sirtuins, PARPs, and other NAD^+^-dependent signaling systems. Therefore, the beneficial effects observed following NR administration represent a cascade of secondary adaptations involving metabolic regulation, stress responses, and cellular resilience. Among the best-supported mechanisms, activation of NAD^+^-dependent sirtuins represents a central pathway linking NR supplementation to mitochondrial and neuronal protection. Increased NAD^+^ availability enhances SIRT1 and SIRT3 activity, which promotes mitochondrial biogenesis, improves oxidative phosphorylation efficiency, regulates antioxidant responses, and maintains mitochondrial quality control through pathways involving peroxisome proliferator-activated receptor gamma coactivator 1-alpha (PGC-1α) and related metabolic regulators (17, 34, 35). These mechanisms are supported predominantly by experimental studies, and their contribution to clinically relevant neuroprotection in humans remains to be established. These effects are particularly relevant in aging neurons, which exhibit high metabolic demands and increased vulnerability to mitochondrial dysfunction. NR-mediated NAD^+^ restoration may also influence genomic stability by supporting NAD^+^-consuming DNA repair enzymes. Although PARP activation is essential for maintaining genome integrity, excessive or chronic PARP activity during aging can accelerate NAD^+^ depletion. Thus, restoring NAD^+^ availability may help rebalance DNA repair capacity while preventing excessive metabolic stress caused by sustained NAD^+^ consumption (17, 81). A growing body of evidence further suggests that NAD^+^ restoration modulates age-associated inflammatory responses. Experimental models indicate that NR supplementation can reduce microglial activation and attenuate inflammatory signaling pathways, including NF-κB-related responses and inflammasome activation. However, the relative contribution of these mechanisms to human brain aging remains uncertain because most evidence derives from animal and cellular models rather than clinical studies. Synaptic maintenance represents another potential mechanism through which NAD^+^ restoration may influence cognitive resilience. NR-induced increases in NAD^+^ availability may indirectly promote synaptic plasticity through enhanced mitochondrial energy supply, improved redox balance, and regulation of neurotrophic pathways such as BDNF signaling. Importantly, NAD^+^ does not directly regulate neurotransmitter release, dendritic spine remodeling, or synaptic protein expression; rather, these processes occur through downstream pathways influenced by NAD^+^-dependent enzymes and metabolic adaptation. Accordingly, evidence for effects on cognition or synaptic function should be interpreted as downstream and largely preclinical rather than as evidence of a direct clinical effect of NR. Additional mechanisms, including regulation of autophagy/mitophagy, insulin–Akt signaling, and endoplasmic reticulum stress responses, have been proposed as contributors to the effects of NAD^+^ restoration during aging. Nevertheless, these pathways remain emerging areas of investigation, and their relative importance compared with the more consistently supported mitochondrial and sirtuin-mediated mechanisms requires further clarification. Collectively, current evidence suggests that NR supplementation acts through an interconnected metabolic network rather than a single therapeutic pathway. Restoration of NAD^+^ availability may improve cellular resilience by coordinating mitochondrial function, genomic maintenance, inflammatory regulation, and synaptic support. However, the majority of mechanistic evidence remains preclinical, emphasizing the need for human studies that directly evaluate whether molecular improvements translate into preserved cognition and reduced neurodegenerative risk.

## 7 Effects of vitamin B3–derived NAD^+^ precursors (niacin, NAM, NR, and NMN) on AD

### 7.1 Preclinical and translational evidence

AD is characterized by progressive impairment of mitochondrial bioenergetics, chronic neuroinflammation, impaired proteostasis, and synaptic vulnerability, processes that are closely associated with age-related NAD^+^ depletion. Preclinical studies suggest that restoration of NAD^+^ availability through vitamin B3-derived precursors may influence multiple interconnected pathways involved in AD pathology, although most evidence remains derived from experimental models and requires further clinical validation. In APP/PS1 transgenic mouse models, niacin supplementation was investigated using transcriptomic profiling and weighted gene co-expression network analysis (WGCNA) to identify molecular pathways associated with cognitive improvement. Niacin administration improved cognitive performance after 6 months of treatment and was associated with modulation of genes involved in synaptic plasticity, circadian regulation, ubiquitin-mediated proteolysis, and long-term potentiation-related pathways (82) (Table 2). These findings indicate that niacin may influence AD-related molecular networks beyond its classical metabolic effects; however, the precise contribution of NAD^+^ restoration versus other niacin-mediated actions remains incompletely defined. A major body of evidence has focused on NR, particularly because of its capacity to increase NAD^+^ availability in aging tissues. In APP/PS1 mice, AD pathology was accompanied by reduced brain NAD^+^ levels, increased activation of microglia and astrocytes, enhanced inflammatory signaling, and greater cellular stress. Long-term NR administration restored NAD^+^ levels and was associated with reduced neuroinflammatory markers, decreased NOD-, LRR- and pyrin domain-containing protein 3 (NLRP3) inflammasome activation, attenuation of DNA damage and cellular senescence, and improvement of synaptic function and cognitive performance. Mechanistically, these effects were linked to modulation of the cGAS–STING pathway, mitochondrial quality control, and inflammatory regulation (83). However, these mechanistic and cognitive effects have been demonstrated primarily in experimental models, and their relevance to human AD remains to be determined. Rather than acting directly on cognition, NR appears to exert neuroprotective effects through downstream regulation of mitochondrial homeostasis, inflammatory signaling, and neuronal resilience.

**TABLE 2:** Effects of NAD^+^-boosting interventions on Alzheimer’s disease pathology: mechanistic insights, translational evidence, and unresolved questions from preclinical studies.

Population/ model	Intervention	Key mechanisms	Main findings	Evidence synthesis and remaining uncertainties	References
APP/PS1 transgenic AD mice	Nicotinic acid (niacin) supplementation	↑ NAD/NADP; modulation of gene networks (Ctnnb1, Mdm2, Crebbp, Gnb2l1, Pten); pathways: circadian rhythm, ubiquitin-proteasome, LTP	↑ cognitive function, ↑ synaptic plasticity (LTP), ↑ gene regulation (neuroprotective pathways)	Transcriptomic and *in vivo* evidence suggests that niacin may influence AD-related cognitive decline through multiple molecular networks rather than a single pathway. However, the specific contribution of NAD^+^ restoration versus other niacin-mediated effects remains unclear.	(82)
APP/PS1 transgenic AD mice	NR supplementation (5 months)	↑ NAD^+^; ↓ neuroinflammation; ↓ cGAS-STING signaling; ↑ mitophagy; ↓ DNA damage and senescence	↑ NAD^+^ levels, ↓ neuroinflammation, ↓ microglia/astrocyte activation, ↓ Aβ pathology, ↓ apoptosis/senescence, ↑ cognitive function	Strong mechanistic evidence indicates that NAD^+^ depletion contributes upstream to neuroinflammation and cellular senescence in AD. However, the relative importance of cGAS-STING inhibition compared with other NAD^+^-dependent pathways requires further clarification.	(83)
Aged mice + APP/PS1 AD mice	NR supplementation (3 months)	↑ NAD^+^; ↓ astrocyte activation; ↓ Aβ accumulation; modulation of NAMPT	↑ memory (spatial and fear), ↓ Aβ accumulation, ↓ astrocyte activation, ↓ NAMPT, ↓ body weight gain	Multiple animal studies consistently demonstrate cognitive and pathological benefits of NR, particularly through mitochondrial and inflammatory mechanisms. Translation to human cognitive outcomes remains uncertain.	(84)
3xTgAD/Polβ^ + ⁣/−^ mice (DNA repair-deficient AD model)	NR supplementation	↑ NAD^+^/NADH ratio; ↑ SIRT3; ↓ DNA damage; ↓ neuroinflammation; improved synaptic plasticity	↑ NAD^+^ levels, ↓ pTau pathology, ↔ Aβ, ↓ DNA damage, ↓ neuroinflammation, ↓ apoptosis, ↑ synaptic plasticity, ↑ cognitive function	Evidence supports NAD^+^ depletion as an upstream driver of multiple AD-related abnormalities. However, NR effects appear stronger in models with DNA repair impairment, suggesting disease-context dependency.	(5)
APP/PS1 transgenic AD mice	NR supplementation	↑ NAD^+^; modulation of gut microbiota (↑ Bacteroides); ↓ oxidative stress; regulation of ferroptosis; ↑ mitochondrial function	↑ gut microbiota balance, ↓ Aβ accumulation, ↓ oxidative stress, ↑ mitochondrial function, ↑ synaptic density, ↑ cognitive function	Emerging evidence expands NAD^+^ biology beyond neuronal mechanisms by implicating microbiome-mediated pathways. The causal relationship between microbiota changes and neuroprotection requires further validation.	(85)
3xTg/POLβ^ + ⁣/−^ AD mice (sex-dependent analysis)	NR supplementation ± ketogenic diet (12 weeks)	Modulation of NAD^+^ redox state; ↑ TCA cycle intermediates; improved mitochondrial bioenergetics (ETC function)	↑ mitochondrial energetics, ↑ TCA intermediates, ↑ ETC efficiency, ↑ NAD^+^ redox balance (sex-dependent effects)	Metabolic interventions may modify NAD^+^-dependent mitochondrial dysfunction in AD; however, biological sex and metabolic background appear important determinants of therapeutic response.	(86)
AD patient samples + AD mouse models	NMN supplementation	↑ NAD^+^; activation of ATF4-dependent mitochondrial UPR (UPRmt); ↑ mitophagy; improved mitochondrial proteostasis	↑ mitochondrial stress response, ↓ protein aggregation, ↓ neuronal loss, ↓ brain atrophy, ↑ synaptic function, ↑ memory performance	Recent findings identify mitochondrial stress adaptation as an important therapeutic target of NAD^+^ enhancement. Whether activation of UPRmt is necessary for clinical benefit remains unknown.	(87)
APP/PS1 AD mice + Aβ-induced PC12 cells	NMN supplementation	↑ NAD^+^; ↑ autophagy; activation of Nrf2/Keap1/NQO1 pathway; ↓ oxidative stress; ↑ p-tau clearance	↑ autophagy, ↓ p-tau, ↓ oxidative stress, ↑ neuronal survival, ↑ cognitive function	Independent studies converge on oxidative stress and impaired proteostasis as major NAD^+^-responsive mechanisms. However, pathway interactions between autophagy and antioxidant signaling need further investigation.	(88)
APP/PS1 AD mice	NR supplementation (8 weeks)	↑ NAD^+^; modulation of gut microbiota diversity; restoration of microbial balance (e.g., *Bacteroides*, *Akkermansia*, *Lactobacillus*)	↑ gut microbiota diversity, ↓ dysbiosis, ↓ neuroinflammation, ↓ cognitive impairment, ↑ gut–brain axis homeostasis	Gut microbiome modulation represents an emerging mechanism linking NAD^+^ metabolism with neurodegeneration. Future studies should determine whether microbiome changes mediate or simply accompany NR-induced neuroprotection.	(89)

AD, Alzheimer’s disease; NR, nicotinamide riboside; NMN, nicotinamide mononucleotide; NAM, nicotinamide; NA, nicotinic acid (niacin); NAD^+^, nicotinamide adenine dinucleotide; NADH, reduced nicotinamide adenine dinucleotide; APP/PS1, amyloid precursor protein/presenilin 1 transgenic mouse model; Polβ, DNA polymerase beta; UPRmt, mitochondrial unfolded protein response; ATF4, activating transcription factor 4; Nrf2, nuclear factor erythroid 2–related factor 2; Keap1, kelch-like ECH-associated protein 1; NQO1, NAD(P)H quinone dehydrogenase 1; cGAS, cyclic GMP-AMP synthase; STING, stimulator of interferon genes; SIRT3, sirtuin 3; SIRT1, sirtuin 1; Aβ, amyloid-beta; pTau, phosphorylated tau; ROS, reactive oxygen species; UPRmt, mitochondrial unfolded protein response; LTP, long-term potentiation. Collectively, preclinical studies indicate that NAD^+^-boosting interventions exert neuroprotective effects in AD models through converging mechanisms involving mitochondrial restoration, DNA repair, suppression of neuroinflammation, enhancement of autophagy, regulation of oxidative stress, and modulation of gut–brain communication. Despite consistent benefits across multiple animal models, important uncertainties remain regarding the translatability of these findings to humans, including optimal intervention type, dosage, treatment duration, disease stage, and the contribution of tissue-specific NAD^+^ metabolism.

Consistent with these findings, another study demonstrated that NR supplementation improved short-term spatial memory in aged mice and contextual fear memory in AD models. These effects were accompanied by reduced microglial and astrocytic activation, decreased amyloid plaque-associated astrocyte recruitment, and attenuation of Aβ pathology (84). Similarly, in 3xTgAD and DNA repair-deficient 3xTgAD/Polβ^ + ⁣/−^ models, impaired NAD^+^/NADH balance and defective DNA repair were associated with exacerbated AD-like pathology. NR administration restored NAD^+^ homeostasis, reduced tau pathology, oxidative damage, neuroinflammation, and synaptic dysfunction, while improving cognitive performance. However, NR did not significantly alter amyloid-β deposition, suggesting that NAD^+^ restoration may preferentially regulate specific pathological pathways rather than universally suppress all AD hallmarks (5). Together, these studies indicate that NR-mediated NAD^+^ replenishment may primarily affect mitochondrial dysfunction, genomic instability, and inflammatory mechanisms, while its effects on amyloid pathology remain model-dependent. Beyond direct neuronal mechanisms, emerging evidence suggests that NAD^+^ precursors may interact with the gut–brain axis in AD. NMN supplementation in APP/PS1 mice improved gut microbial composition, enhanced beneficial bacterial populations, restored intestinal barrier integrity, and altered microbiota-associated metabolic profiles. These peripheral changes were accompanied by improved mitochondrial function, increased hippocampal synaptic density, reduced oxidative stress, decreased amyloid accumulation, and enhanced cognitive performance (85). Although these findings support a potential microbiota-mediated component of NAD^+^ modulation, the causal relationship between microbial alterations and brain outcomes remains to be established.

Metabolic interventions combining NAD^+^ precursors with dietary approaches have also provided mechanistic insights. In 3xTgAD/Polβ^ + ⁣/−^ mice, NR supplementation combined with ketogenic dietary intervention altered hippocampal mitochondrial metabolism, NAD^+^/NADH redox balance, and TCA cycle intermediates. These effects were influenced by sex and metabolic background, highlighting the importance of biological heterogeneity when evaluating NAD^+^-based interventions (86). Furthermore, NMN administration improved mitochondrial proteostasis by activating ATF4-dependent mitochondrial unfolded protein response, enhancing mitochondrial quality control, reducing neuronal loss, and preserving hippocampal synaptic integrity in AD models. Transcriptomic and proteomic analyses suggested that astrocytes may represent important cellular mediators of NMN-induced mitochondrial adaptation (87). Additional experimental evidence indicates that NMN may regulate oxidative stress and protein aggregation pathways. In cellular and animal AD models, NMN increased NAD^+^ availability, enhanced autophagic flux, promoted phosphorylated tau clearance, and activated antioxidant responses through the Nrf2/Keap1/NQO1 pathway. Pharmacological inhibition of autophagy or Nrf2 signaling reduced these protective effects, supporting the involvement of these pathways in NMN-mediated neuroprotection (88). The interaction between NAD^+^ precursors and gut microbiota has also been investigated using NR supplementation. In APP/PS1 mice, NR administration partially restored microbiome diversity and modified disease-associated microbial alterations, including increased abundance of potentially beneficial taxa such as *Bifidobacterium*, *Akkermansia*, *Lactobacillus*, and *Bacteroides*. These microbiome changes were associated with reduced inflammatory signaling and improved cognitive-related outcomes; however, whether microbiota remodeling represents a cause or consequence of NAD^+^-mediated neuroprotection remains unresolved (89).

Collectively, preclinical evidence indicates that vitamin B3-derived NAD^+^ precursors may improve AD-related outcomes through converging mechanisms involving mitochondrial restoration, inflammatory regulation, DNA repair capacity, proteostasis, and gut–brain communication. Nevertheless, these effects are predominantly demonstrated in transgenic animal models, and the extent to which NAD^+^ augmentation can prevent or modify human AD progression remains uncertain. Accordingly, these findings provide a mechanistic rationale for further clinical investigation but do not yet establish NAD^+^ precursor supplementation as an effective disease-modifying treatment for AD.

### 7.2 Critical appraisal of current evidence on vitamin B3–derived NAD^+^ precursor supplementation in AD

Despite consistent mechanistic findings from experimental models, the current evidence supporting vitamin B3-derived NAD^+^ precursor supplementation as a potential disease-modifying approach for AD remains preliminary (5, 24). Most available studies have been conducted in transgenic mouse models, particularly APP/PS1, 3xTgAD, and DNA repair-deficient AD models, which provide valuable mechanistic insights but do not fully reproduce the complexity of human AD pathology (5, 72). These models generally involve relatively small experimental groups and short-to-medium intervention periods (approximately 3–6 months), limiting conclusions regarding long-term efficacy, safety, and preventive potential. Human evidence remains considerably more limited. Available clinical studies involving NR, NMN, or related NAD^+^-enhancing interventions have primarily focused on safety, pharmacokinetics, and biological target engagement rather than definitive cognitive or disease-modifying outcomes (71, 74). Although several trials have demonstrated increased circulating NAD^+^ metabolites and favorable changes in selected biomarkers, including inflammatory or metabolic indicators, these molecular alterations have not consistently translated into measurable improvements in cognition, functional independence, or neurodegenerative progression (35, 71). This discrepancy highlights an important translational gap between successful NAD^+^ restoration and clinically meaningful neurological benefits. Several factors may contribute to the heterogeneity of reported outcomes. Differences in precursor type (NR, NMN, NAM, or niacin), dosage, treatment duration, disease stage, genetic background, sex, dietary conditions, and baseline metabolic status may substantially influence therapeutic responses (27, 35, 76). Furthermore, many studies rely on surrogate markers, such as NAD^+^ concentrations, inflammatory mediators, mitochondrial parameters, or amyloid/tau-related biomarkers, which may not accurately predict long-term cognitive outcomes (71). Future investigations should therefore prioritize clinically relevant endpoints, including longitudinal cognitive trajectories, functional outcomes, and validated neurodegenerative biomarkers. The cellular specificity of NAD^+^ interventions also requires further clarification. Neurons, astrocytes, microglia, and oligodendrocytes exhibit distinct metabolic demands and NAD^+^-related enzyme expression profiles, which may result in cell-specific responses to NAD^+^ augmentation. For example, astrocyte-mediated mitochondrial adaptation and microglial inflammatory regulation have emerged as potential contributors to NAD^+^-dependent neuroprotection; however, their relative contribution compared with direct neuronal effects remains insufficiently characterized (5).

Another important consideration is the relationship between NAD^+^ restoration and disease stage. Current evidence suggests that NAD^+^ supplementation may be more likely to exert beneficial effects in conditions characterized by metabolic stress, mitochondrial impairment, or early cellular dysfunction rather than advanced neurodegeneration with extensive neuronal loss (5, 24). Therefore, NAD^+^ enhancement could potentially be investigated not only as an adjunctive approach in established AD pathology but also as a preventive or risk-reduction strategy during aging before irreversible neuronal damage occurs. Finally, although emerging studies indicate that vitamin B3-derived precursors may influence gut microbiota composition and gut–brain communication, the causal mechanisms linking microbiome remodeling with NAD^+^-mediated neuroprotection remain unclear (24). Future studies integrating metabolomics, microbiome profiling, neuroimaging, and longitudinal clinical assessments will be essential to determine whether microbiota-targeted approaches can enhance the efficacy of NAD^+^-based interventions. Overall, current evidence supports NAD^+^ precursor supplementation as a biologically plausible metabolic approach capable of modulating multiple AD-related pathways; however, translation into clinical practice requires larger, longer, well-controlled human trials addressing optimal dosing, treatment timing, long-term safety, patient stratification, and meaningful neurological outcomes. Importantly, the observed benefits of NAD^+^ augmentation in experimental studies appear to be strongly dependent on disease stage and cellular context. Experimental studies indicate that restoration of NAD^+^ metabolism may be particularly effective during early phases of metabolic dysfunction, mitochondrial impairment, or DNA repair deficiency, whereas its impact may be limited once extensive neuronal loss and irreversible pathological changes have occurred. Therefore, future clinical applications should consider NAD^+^ status, metabolic phenotype, and disease stage as potential determinants of therapeutic responsiveness rather than assuming a universal benefit across all individuals with AD (5, 90).

### 7.3 Molecular mechanisms of NAD^+^ repletion in AD

Age-associated NAD^+^ depletion is increasingly recognized as a converging metabolic disturbance that may link mitochondrial dysfunction, impaired stress responses, neuroinflammation, and synaptic vulnerability in AD. However, NAD^+^ precursors do not directly regulate cognitive processes; rather, their effects are mediated through downstream NAD^+^-dependent signaling networks that influence neuronal and glial function. Restoration of NAD^+^ availability through NR, NMN, and NAM supplementation increases substrate availability for NAD^+^-dependent enzymes, particularly sirtuins, PARPs, and CD38-related pathways, thereby influencing multiple cellular processes implicated in AD progression. A major mechanism involves the activation of NAD^+^-dependent sirtuins, particularly SIRT1 and SIRT3, which regulate mitochondrial quality control, oxidative metabolism, and cellular stress resistance. Increased SIRT1 activity may promote mitochondrial biogenesis, regulate inflammatory transcriptional responses, and support synaptic maintenance through downstream modulation of pathways associated with neuroplasticity. Similarly, SIRT3 contributes to mitochondrial antioxidant defense by regulating enzymes involved in ROS detoxification and maintaining mitochondrial homeostasis. Thus, the available evidence suggests that NAD^+^ restoration may support neuronal resilience primarily through sirtuin-mediated downstream adaptations rather than through direct regulation of synaptic function.

Mitochondrial dysfunction represents another central mechanism connecting NAD^+^ depletion with AD pathology. Reduced NAD^+^ availability compromises oxidative phosphorylation, disrupts NAD^+^/NADH redox balance, and increases mitochondrial oxidative stress, thereby contributing to neuronal energy failure. NAD^+^ precursor supplementation has been associated with improved mitochondrial respiration, enhanced mitophagy, and restoration of mitochondrial quality control mechanisms in experimental AD models. In particular, NMN-mediated activation of mitochondrial unfolded protein response and NR-associated improvements in mitochondrial metabolic flexibility suggest that NAD^+^ availability may contribute to mitochondrial adaptation during cellular stress.

NAD^+^ restoration also appears to influence neuroinflammatory pathways, which represent a major driver of AD progression. Experimental studies indicate that NR and NMN supplementation can reduce microglial activation and inflammatory signaling through modulation of pathways including NF-κB, NLRP3 inflammasome, and cGAS–STING signaling. These findings suggest that the anti-inflammatory effects associated with NAD^+^ enhancement are largely mediated through regulation of immune-cell activation and stress-response pathways rather than direct suppression of inflammation by NAD^+^ itself. The interaction between NAD^+^ metabolism and CD38 activity is particularly relevant, as age-associated increases in CD38 expression in immune and glial cells may accelerate NAD^+^ consumption and amplify inflammatory states. Maintenance of genomic stability and proteostasis represents another important mechanism. Chronic DNA damage during aging increases PARP activation, leading to excessive NAD^+^ consumption and further metabolic impairment. NAD^+^ replenishment may therefore support cellular capacity for DNA repair while potentially limiting excessive energetic depletion. In parallel, activation of autophagic and mitophagic pathways facilitates removal of damaged proteins and mitochondria, processes that are essential for maintaining neuronal integrity in AD.

Synaptic dysfunction and dendritic spine degeneration are among the earliest pathological features associated with cognitive decline. Although NAD^+^ does not directly regulate neurotransmitter release, postsynaptic density proteins, or dendritic remodeling, NAD^+^-dependent pathways may indirectly influence synaptic plasticity through mitochondrial support, SIRT1-mediated transcriptional regulation, BDNF-related signaling, and improved neuronal energy availability. This distinction is important because preservation of synaptic structure is more appropriately interpreted as a downstream consequence of improved cellular resilience rather than a direct action of NAD^+^ molecules. Emerging evidence also suggests that NAD^+^ metabolism interacts with the gut–brain axis in AD. NAD^+^ precursor supplementation may influence microbiota composition, intestinal barrier function, and microbiota-derived metabolites, which could subsequently affect neuroinflammation, metabolic regulation, and neuronal function. However, whether microbiome alterations are necessary mediators of NAD^+^-induced neuroprotection or secondary consequences of improved systemic metabolism remains unresolved. Collectively, experimental evidence suggests that NAD^+^ repletion in AD may operate through an interconnected network involving sirtuin activation, mitochondrial adaptation, inflammatory regulation, DNA repair, proteostasis, synaptic preservation, and gut–brain communication. Among these mechanisms, mitochondrial quality control, neuroimmune regulation, and maintenance of neuronal resilience currently represent the more consistently supported pathways, whereas microbiome-mediated effects and cell-specific responses remain emerging areas requiring further investigation (Figure 2).

**FIGURE 2:**
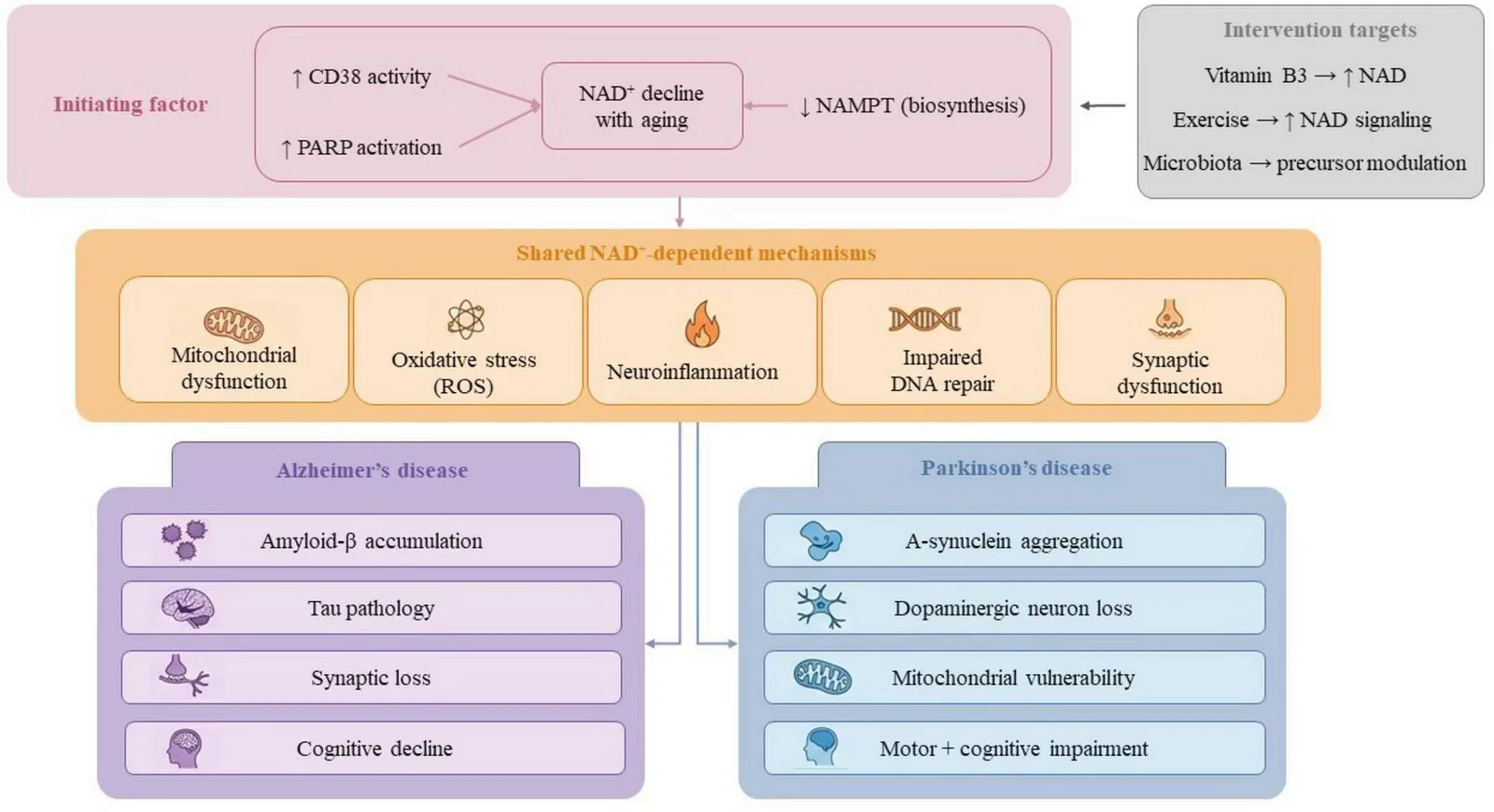
Shared and disease-specific NAD^+^-related mechanisms in neurodegenerative disorders. Age-associated decline in NAD^+^ availability, driven by increased consumption and reduced biosynthesis, contributes to a set of common pathological mechanisms, including mitochondrial dysfunction, oxidative stress, impaired DNA repair, neuroinflammation, and synaptic dysfunction. These shared processes subsequently diverge into disease-specific pathological features, such as amyloid-(accumulation and tau pathology in Alzheimer’s disease, and (-synuclein aggregation and dopaminergic neuronal loss in Parkinson’s disease. NAD^+^ primarily exerts its effects indirectly through downstream signaling pathways rather than acting as a direct effector, thereby influencing both shared and disease-specific aspects of neurodegeneration. NAD^+^, nicotinamide adenine dinucleotide; SIRT, sirtuin; PARP, poly(ADP-ribose) polymerase; CD38, cluster of differentiation 38; ROS, reactive oxygen species; AD, Alzheimer’s disease; PD, Parkinson’s disease.

## 8 Effects of vitamin B3–derived NAD^+^ precursors (niacin, NAM, NR, and NMN) on PD

### 8.1 Preclinical and translational evidence

PD is characterized by selective vulnerability of dopaminergic neurons, particularly within the substantia nigra, where mitochondrial dysfunction, oxidative stress, impaired proteostasis, and α-synuclein accumulation contribute to progressive neuronal loss. Because NAD^+^ metabolism is essential for mitochondrial energy regulation and cellular stress adaptation, NAD^+^ precursor supplementation has emerged as a potential metabolic approach for supporting neuronal resilience in PD. However, current evidence remains predominantly preclinical, and clinical translation is still limited.

Clinical studies have primarily focused on the safety, metabolic engagement, and biological effects of NAD^+^ precursors. In a randomized, double-blind, placebo-controlled phase I trial involving individuals with early-stage PD, NR supplementation (1,000 mg/day for 30 days) was well tolerated and significantly increased cerebral NAD^+^ concentrations measured by magnetic resonance spectroscopy. NR treatment was also associated with alterations in cerebrospinal fluid metabolites, improved mitochondrial and lysosomal gene expression profiles, and reductions in selected inflammatory markers. Participants with greater NAD^+^ elevation showed favorable metabolic changes on PET imaging and modest changes in selected clinical measures, suggesting that NAD^+^ augmentation is biologically active in the human PD brain (91). Nevertheless, larger and longer trials are required to determine whether these molecular effects translate into sustained clinical benefits. Additional clinical evidence has examined the effects of niacin supplementation in PD. In a randomized placebo-controlled trial, low-dose niacin did not significantly improve UPDRS III motor scores after 6 months; however, longer exposure over 12 months was associated with gradual improvement in motor severity and was generally well tolerated (92). Because the primary 6-month motor outcome was not significantly improved, the longer-term findings should be interpreted cautiously and require confirmation in larger controlled studies. Furthermore, NR supplementation did not induce major disturbances in global DNA methylation patterns in patients with PD, including individuals carrying MTHFR variants, suggesting that short-term high-dose NR does not substantially disrupt methylation homeostasis (93) (Table 3). Together, clinical findings support the biological activity and short-term tolerability of NAD^+^ precursors but provide insufficient evidence for definitive disease modification.

**TABLE 3:** Effects of NAD^+^-boosting interventions on Parkinson’s disease: mechanistic insights, translational evidence, and unresolved questions from preclinical and clinical studies.

Population/ model	Intervention	Key mechanisms	Main findings	Evidence synthesis and remaining uncertainties	References
Parkinson’s disease patients (double-blind, placebo-controlled trial, *n* = 29)	Nicotinamide riboside (NR), short-term (≤30 days)	↑ NAD^+^ metabolism; assessment of one-carbon/methylation pathways	↔ DNA methylation homeostasis, ↔ methylation stability (no adverse epigenetic effects)	Clinical evidence supports the short-term safety of NR regarding epigenetic stability. However, long-term effects of chronic NAD^+^ enhancement on methylation-dependent pathways remain unknown.	(93)
Newly diagnosed, treatment-naïve PD patients (randomized phase I trial, *n* = 30)	NR (1,000 mg/day, 30 days)	↑ NAD^+^ in brain; ↑ mitochondrial/lysosomal/ proteasomal pathways; ↓ inflammation; ↑ cerebral metabolism	↑ brain NAD^+^, ↓ inflammatory cytokines, ↑ mitochondrial/lysosomal function, ↑ cerebral metabolism, ↔/↑ mild clinical improvement	The strongest early human evidence indicates that NR can increase brain NAD^+^ availability and modify disease-relevant metabolic pathways. Larger phase II/III trials are required to determine clinical efficacy.	(91)
MPP^+^-treated neuroblastoma cells + α-synuclein transgenic Drosophila	High-dose nicotinamide (vitamin B3)	↑ NAD(P)H antioxidant capacity; ↓ oxidative stress; ↑ mitochondrial complex I activity; ↓ DNA/protein oxidation	↓ oxidative stress, ↑ mitochondrial complex I activity, ↓ DNA damage, ↑ motor function (fly climbing ability)	Preclinical evidence supports NAM-mediated mitochondrial protection as a neuroprotective mechanism; however, effective doses may exceed physiologically achievable concentrations in humans.	(94)
MPTP-induced PD mice	Nicotinamide (NAM, 500 mg/kg)	↑ Nrf2/HO-1 antioxidant pathway; ↓ TLR4/NF-κB inflammatory signaling; ↓ oxidative stress	↓α-synuclein, ↑ TH/DAT levels, ↓ neuroinflammation, ↓ oxidative stress, ↑ motor function	Animal studies consistently indicate antioxidant and anti-inflammatory effects of NAM. Translation to human PD requires evaluation of optimal dosing and long-term safety.	(95)
Parkinson’s disease patients (RCT, *n* = 47)	Low-dose niacin (6–12 months)	Modulation of NAD^+^ metabolism; improved neuronal function and behavioral outcomes	↓ UPDRS III motor scores (improved motor function), ↑ sleep and cognitive-related outcomes (trend), ↔ good tolerability	Human clinical evidence suggests possible symptomatic benefits of niacin as an adjunct therapy, but evidence remains preliminary due to limited sample size and modest effect sizes.	(92)
PC12 cells (rotenone-induced PD model)	Nicotinamide mononucleotide (NMN)	↑ NAD^+^ and ATP production; ↓ apoptosis; improved mitochondrial energy metabolism	↑ cell survival, ↑ NAD^+^/ATP levels, ↓ apoptosis, ↓ LDH release, ↑ mitochondrial function	Cellular evidence demonstrates that NAD^+^ restoration protects against mitochondrial toxin-induced neuronal injury; validation in animal and human studies is needed.	(97)
Proteasome inhibition mouse model + C. elegans α-syn model	Nicotinamide riboside (NR)	↑ mitochondrial function; activation of UPRmt; interaction with proteasome pathways	↑ early motor function (C. elegans and mice), ↓α-syn toxicity, but ↓ dopamine metabolism and ↓ dopaminergic gene expression with long-term treatment	Evidence highlights potential context-dependent effects of NR. While mitochondrial activation may be beneficial, prolonged NAD^+^ enhancement may have unexpected effects on dopaminergic pathways.	(96)
6-OHDA-induced PD mouse model + PC12 cells	Nicotinamide riboside (NR)	↑ mitochondrial function; ↓ oxidative stress; ↓ neuronal damage	↓ motor deficits, ↓ dopaminergic neuron loss, ↓ oxidative stress, ↓ mitochondrial dysfunction	Preclinical evidence supports NAD^+^ depletion as a contributor to PD pathology; however, direct supplementation strategies require further pharmacological optimization.	(98)
MPTP-induced PD zebrafish model	Nicotinamide riboside (NR)	↑ NAD^+^; modulation of glucose metabolism; ↓ ER stress via PERK–eIF2α–ATF4–CHOP pathway	↑ survival, ↑ motor function, ↑ dopamine neurons, ↓ ER stress, ↑ glucose metabolism balance	Emerging evidence suggests that NR may influence PD through metabolic and proteostasis pathways beyond mitochondrial regulation. The relevance of these mechanisms in mammalian PD remains to be determined.	(99)

PD, Parkinson’s disease; NR, nicotinamide riboside; NAM, nicotinamide; NAD^+^, nicotinamide adenine dinucleotide; NAD, nicotinamide adenine dinucleotide (reduced/total form as used in studies); MPTP, 1-methyl-4-phenyl-1,2,3,6-tetrahydropyridine; 6-OHDA, 6-hydroxydopamine; MPP^+^, 1-methyl-4-phenylpyridinium; α-syn, alpha-synuclein; TH, tyrosine hydroxylase; DAT, dopamine transporter; Nrf2, nuclear factor erythroid 2–related factor 2; HO-1, heme oxygenase-1; TLR4, toll-like receptor 4; NF-κB, nuclear factor kappa B; COX-2, cyclooxygenase-2; UPRmt, mitochondrial unfolded protein response; PERK, protein kinase R-like endoplasmic reticulum kinase; eIF2α, eukaryotic initiation factor 2 alpha; ATF4, activating transcription factor 4; CHOP, C/EBP homologous protein; ROS, reactive oxygen species; ATP, adenosine triphosphate; UPRmt, mitochondrial unfolded protein response. Overall, studies investigating NAD^+^-boosting interventions in Parkinson’s disease demonstrate convergent neuroprotective mechanisms involving mitochondrial restoration, oxidative stress reduction, suppression of neuroinflammation, improvement of proteostasis, and regulation of cellular energy metabolism. Human studies, particularly the NADPARK trial, provide initial evidence that NR can increase cerebral NAD^+^ availability and modulate disease-relevant metabolic pathways. However, clinical efficacy remains uncertain because current trials are limited by small sample sizes, short intervention periods, and heterogeneous outcomes. Moreover, emerging evidence suggests that responses to NAD^+^ enhancement may depend on disease stage, intervention type, and duration, emphasizing the need for precision approaches in future PD studies.

Preclinical studies provide stronger mechanistic evidence. In cellular and Drosophila models of PD, NAM preserved mitochondrial complex I activity, reduced ROS accumulation, protected against oxidative DNA and protein damage, and improved motor performance in α-synuclein models (94). Similarly, in MPTP-induced PD mice, NAM preserved dopaminergic neurons by improving NAD^+^-dependent redox balance, activating antioxidant defenses through Nrf2/HO-1 signaling, and reducing inflammatory responses involving TLR4/NF-κB pathways (95). These findings suggest that NAM may support dopaminergic neuronal resilience in experimental models, primarily through improved mitochondrial function and regulation of cellular stress.

NR and NMN have also demonstrated protective effects in toxin-induced and genetic models of PD. NR supplementation improved mitochondrial function, reduced neuronal stress, and enhanced proteostasis in *C. elegans* models expressing α-synuclein through activation of mitochondrial unfolded protein response pathways. However, in a proteasome-impaired mouse model, prolonged NR administration produced adverse effects, including reduced dopamine levels and altered dopaminergic gene expression, despite initial improvements in motor performance (96). This finding highlights that NAD^+^ enhancement may not exert universally beneficial effects and that disease context, duration of treatment, and underlying cellular stress pathways may determine therapeutic outcomes. Other experimental models further support the involvement of NAD^+^ precursors in mitochondrial and metabolic regulation. NMN protected rotenone-treated neuronal cells by restoring intracellular NAD^+^ levels, improving ATP production, reducing mitochondrial damage, and limiting apoptosis (97). Similarly, direct NAD supplementation protected dopaminergic neurons in 6-OHDA models by improving mitochondrial function and reducing oxidative injury (98). In zebrafish models of MPTP-induced PD, NR improved survival, motor behavior, and dopaminergic neuron integrity through regulation of glucose metabolism and attenuation of ER stress pathways, including PERK–eIF2α–ATF4–CHOP signaling (99). Collectively, preclinical evidence indicates that NAD^+^ precursors may support dopaminergic neuronal integrity in experimental PD models through converging mechanisms involving mitochondrial quality control, oxidative stress regulation, proteostasis maintenance, and inflammatory modulation. Nevertheless, compared with AD, evidence in PD remains less extensive, and whether these experimental effects translate into clinically meaningful or disease-modifying benefits in patients with PD remains uncertain and requires validation in larger, adequately powered, longer-term human studies.

### 8.2 Critical appraisal of current evidence on vitamin B3–derived NAD^+^ precursors supplementation in PD

The current evidence supporting NAD^+^ precursor supplementation in PD remains mechanistically promising but clinically preliminary. Most available studies are derived from preclinical models, including toxin-induced models (MPTP and 6-OHDA), transgenic organisms, zebrafish, Drosophila, and cellular systems. Although these models have provided important insights into mitochondrial dysfunction, oxidative stress, proteostasis impairment, and neuroinflammatory mechanisms associated with NAD^+^ depletion, their ability to reproduce the progressive and multifactorial nature of idiopathic PD remains limited. In particular, toxin-based models primarily represent acute dopaminergic injury rather than the chronic interaction among aging, genetic susceptibility, environmental factors, and systemic metabolic alterations observed in human PD.

Clinical evidence is currently restricted to early-phase studies with relatively small cohorts and short intervention periods. Although NR supplementation has demonstrated acceptable safety profiles and biological target engagement, including increased NAD^+^ availability and alterations in metabolic or inflammatory biomarkers, consistent improvements in clinical outcomes, including motor symptoms, cognition, or disease progression, have not yet been established. Similarly, studies evaluating niacin have suggested potential long-term benefits; however, the absence of robust placebo-controlled evidence with sufficiently powered samples prevents definitive conclusions regarding therapeutic efficacy. A major challenge is the discrepancy between strong mechanistic effects observed in experimental models and limited functional improvements reported in human studies. Several investigations demonstrate restoration of NAD^+^ levels, enhanced mitochondrial function, reduced oxidative stress, or modulation of inflammatory pathways; however, these molecular changes do not necessarily translate into clinically meaningful neuroprotection.

Furthermore, heterogeneity in precursor type (NR, NMN, NAM, or niacin), dosage, treatment duration, disease stage, and outcome measurements substantially complicates comparisons among studies. Importantly, recent findings suggest that NAD^+^ augmentation may not exert uniformly beneficial effects under all pathological conditions. The response to NAD^+^ restoration appears to depend on cellular context, including mitochondrial status, proteostasis capacity, disease stage, and the balance between metabolic recovery and pathway-specific adaptations. For example, while increased NAD^+^ availability may enhance mitochondrial resilience in some models, excessive or prolonged supplementation under conditions of impaired proteasomal function may adversely influence dopaminergic signaling, highlighting the necessity of defining optimal therapeutic windows. Future investigations should prioritize larger randomized controlled trials with longer follow-up periods, standardized dosing strategies, and clinically relevant endpoints. In addition, integrating NAD^+^ supplementation with complementary interventions, such as exercise and microbiome-targeted approaches, may provide a potentially complementary strategy by simultaneously addressing metabolic dysfunction, inflammation, and neuronal resilience.

Overall, NAD^+^ precursors represent a biologically plausible approach that warrants further in PD; however, current evidence supports biological plausibility rather than confirmed clinical efficacy. A critical consideration emerging from current evidence is that NAD^+^ restoration may not produce identical outcomes across different PD models. While mitochondrial dysfunction and oxidative stress represent shared pathological features, the response to NAD^+^ augmentation appears to depend on the underlying mechanism of neuronal injury. For example, toxin-induced models primarily reflect acute mitochondrial damage, whereas genetic or proteostasis-related models may better capture chronic mechanisms of PD progression. This heterogeneity emphasizes the need for mechanism-specific clinical strategies and biomarker-guided patient selection before NAD^+^-targeted interventions can be appropriately evaluated for clinical use (77, 100).

### 8.3 Molecular mechanisms of NAD^+^ repletion in PD

NAD^+^ precursor supplementation in PD models has been reported to support cellular metabolic capacity and neuronal resilience rather than through direct regulation of individual pathological pathways. NR, NMN, NAM, and niacin increase NAD^+^ availability, thereby supporting NAD^+^-dependent enzymatic reactions involved in mitochondrial metabolism, redox regulation, and cellular stress responses. However, the observed effects of NAD^+^ restoration are thought to be largely mediated through downstream pathways, including sirtuin activation, mitochondrial quality control, antioxidant responses, and inflammatory regulation. In dopaminergic neurons affected by PD-related stress, NAD^+^ replenishment has been associated with improved mitochondrial bioenergetics by enhancing the NAD^+^/NADH balance, supporting oxidative phosphorylation, and promoting mitochondrial quality control mechanisms. Activation of NAD^+^-dependent sirtuins, particularly SIRT1 and SIRT3, contributes to mitochondrial maintenance through regulation of mitochondrial biogenesis, metabolic adaptation, and stress resistance. These effects have been linked to downstream activation of pathways such as PGC-1α-mediated mitochondrial regulation; however, the extent to which this mechanism contributes to human PD progression remains to be determined.

NAD^+^ restoration may also attenuate oxidative and inflammatory stress, two major contributors to dopaminergic vulnerability. Increased NAD^+^ availability supports NADPH-dependent antioxidant capacity and may enhance endogenous defense systems, including Nrf2-mediated antioxidant signaling. In parallel, NAD^+^ precursors have been reported to modulate neuroinflammatory activation by influencing pathways involved in microglial and astrocytic responses, including TLR4/NF-κB and NLRP3 inflammasome signaling. Nevertheless, whether these anti-inflammatory effects represent primary mechanisms of neuroprotection or secondary consequences of improved mitochondrial function remains unresolved. Furthermore, NAD^+^ supplementation has been reported to influence mechanisms involved in neuronal maintenance, including mitochondrial unfolded protein response, mitophagy, autophagy, and proteostasis regulation. These adaptations may contribute to reduced α-synuclein toxicity and improved neuronal survival in experimental PD models. Emerging evidence also suggests that NAD^+^-dependent regulation of endoplasmic reticulum stress, particularly through the PERK–eIF2α–ATF4–CHOP pathway, may represent an additional mechanism linking metabolic restoration with neuronal stress resistance.

Collectively, available evidence indicates that NAD^+^ restoration in PD may influence an interconnected network involving mitochondrial function, oxidative stress control, inflammation regulation, and proteostasis maintenance. However, these mechanisms should be interpreted cautiously because most evidence originates from experimental models, and the relative contribution of each pathway to human PD remains unclear. Future studies should determine whether NAD^+^ enhancement can meaningfully influence disease progression, preserve dopaminergic circuitry, and improve functional outcomes beyond short-term metabolic improvements.

## 9 Effects of vitamin B3–derived NAD^+^ precursors combined with exercise on aging and skeletal muscle function

### 9.1 Preclinical and clinical evidence

NAD^+^ precursor supplementation combined with exercise has emerged as a potential strategy to influence metabolic adaptation during aging; however, current evidence remains limited and heterogeneous. Human studies have primarily focused on short-term physiological outcomes rather than clinically meaningful improvements in aging-related functional decline. A randomized pilot trial in sedentary older adults with hypertension examined whether NR supplementation could enhance the cardiovascular adaptations induced by aerobic exercise. Although combined NR and exercise did not significantly reduce daytime systolic blood pressure compared with exercise alone, NR increased NAD^+^ metabolites and showed trends toward improved vascular parameters, suggesting biological target engagement without clear additive clinical benefits (101) (Table 4). Similarly, supplementation with NAD^+^ precursors containing tryptophan, nicotinic acid, and NAM failed to improve skeletal muscle mitochondrial respiration or exercise efficiency in older adults with reduced physical performance. Despite increased methyl-nicotinamide levels, indicating altered NAD^+^ metabolism, functional outcomes remained unchanged, highlighting the difference between metabolic activation and physiological improvement (102). Acute NR administration has demonstrated some favorable metabolic effects under conditions of age-related metabolic vulnerability. In older men, NR improved NAD(P)H availability, reduced oxidative stress markers, and enhanced muscle strength, whereas comparable effects were not observed in younger individuals (103). These findings suggest that baseline metabolic status may influence responsiveness to NAD^+^ augmentation. Preclinical studies provide stronger evidence for an interaction between NAD^+^ precursors and exercise. NAM supplementation enhanced SIRT1 activity and mitochondrial adaptation in aged skeletal muscle, although combined NAM and exercise produced context-dependent responses, indicating that NAD^+^ interventions may not uniformly amplify exercise adaptations (104). In aged mice, NMN combined with aerobic exercise was associated with greater improvements in muscle strength, endurance, oxygen consumption, glucose tolerance, and metabolic efficiency than either intervention alone, accompanied by increased NAMPT and SIRT1 expression (105). However, the link between NAD^+^ repletion and functional outcomes in skeletal muscle is still complex. New evidence suggests that depletion of NAD^+^ in skeletal muscle does not inevitably cause clear functional deterioration or accelerate aging-related phenotypes, indicating that the effects of NAD^+^ reduction may vary with tissue context, metabolic requirements, and compensatory responses. Together, these results underscore a key difference between skeletal muscle and neuronal tissues: skeletal muscle may exhibit distinct consequences of NAD^+^ dysregulation due to energetic vulnerability, restricted regenerative capacity, and a strong reliance on mitochondrial homeostasis. Consequently, while increasing NAD^+^ levels may influence metabolic resilience in aging muscle, its benefits should not be presumed to translate directly into neuroprotective effects. Additional research is required to determine whether NAD^+^-driven adaptations in peripheral tissues can indirectly support brain health through systemic metabolic control, regulation of inflammation, or exercise-mediated signaling pathways (18).

**TABLE 4:** Effects of NAD^+^-boosting interventions combined with exercise on aging-related metabolic and physical outcomes: mechanistic insights, translational evidence, and remaining challenges.

Population/ model	Intervention	Key mechanisms	Main findings	Evidence synthesis and remaining uncertainties	Study
Sedentary middle-aged and older adults (≥55 years) with hypertension	NR (1,000 mg/day) + aerobic exercise (6 weeks)	↑ NAD metabolism; vascular function modulation; arterial stiffness (PWV) pathways	↔ systolic BP reduction vs exercise alone, ↑ NAD metabolites, ↓ PWV trend, ↑ nighttime BP reduction trend (subgroup)	Early clinical evidence suggests that NAD^+^ enhancement may complement exercise-related vascular adaptations but does not consistently improve blood pressure outcomes. Larger and longer-duration trials are needed to identify responsive populations and optimal combinations.	(101)
Physically impaired older adults (∼73 years)	Niacin equivalents (tryptophan, nicotinic acid, nicotinamide) for 32 days	↑ NAD^+^ metabolism; mitochondrial bioenergetics (skeletal muscle)	↔ skeletal muscle NAD^+^, ↔ mitochondrial respiration, ↔ exercise efficiency, ↑ methyl-nicotinamide (metabolic activation)	Human evidence indicates that increasing NAD^+^-related metabolites does not necessarily translate into improved mitochondrial or functional outcomes. Tissue-specific NAD^+^ regulation and intervention duration may influence efficacy.	(102)
Young vs. old men (double-blind crossover study)	Acute NR supplementation	↑ NAD(P)H redox balance; ↓ oxidative stress; improved antioxidant capacity	↑ NAD(P)H, ↓ oxidative stress markers, ↑ isometric strength, ↑ fatigue resistance (only in older adults), ↔ VO_2_max	Evidence suggests that NAD^+^ precursors may provide greater functional benefits in individuals with age-associated redox imbalance rather than healthy younger populations, supporting a deficiency-dependent response model.	(103)
Young (5 months) and aged (28 months) rats	Nicotinamide (0.5%–1% in drinking water) + treadmill exercise (4 weeks)	↑ NAD^+^ availability; modulation of SIRT1 activity; ↑ PGC-1α; ↑ CREB signaling	↑ SIRT1 activity (functional), ↑ PGC-1α, ↑ mitochondrial biogenesis signaling, ↔/↓ SIRT1 under combined stress in aged muscle	Preclinical findings highlight potential benefits of NAM for skeletal muscle aging but also indicate that NAD^+^ precursors may interact with exercise-induced signaling in a dose- and age-dependent manner.	(104)
Aged C57BL/6J mice (85 weeks)	NMN (300 mg/kg/day) + aerobic exercise (6 weeks)	↑ NAD^+^ salvage pathway; ↑ NAMPT; ↑ SIRT1; ↑ mitochondrial metabolism; improved glucose handling	↑ muscle strength, ↑ endurance, ↑ aerobic capacity, ↑ glucose tolerance, ↑ NAMPT/SIRT1 expression, ↑ energy metabolism	Animal evidence supports synergistic effects between NAD^+^ enhancement and exercise on metabolic health and physical performance. However, translation to humans requires confirmation due to differences in dose, metabolism, and exercise response.	(105)

NR, nicotinamide riboside; NMN, nicotinamide mononucleotide; NAM, nicotinamide; NAD^+^, nicotinamide adenine dinucleotide; NAMPT, nicotinamide phosphoribosyltransferase; SIRT1, sirtuin 1; PGC-1α, peroxisome proliferator-activated receptor gamma coactivator 1-alpha; CREB, cAMP response element-binding protein; p53, tumor protein p53; PARP, poly(ADP-ribose) polymerase; VO_2_max, maximal oxygen uptake; OGTT, oral glucose tolerance test. Collectively, available evidence suggests that NAD^+^-boosting interventions may enhance exercise adaptations through converging mechanisms involving mitochondrial bioenergetics, redox regulation, SIRT1/PGC-1α signaling, and metabolic flexibility. However, responses appear highly dependent on age, baseline NAD^+^ availability, intervention type, and physiological context. While preclinical studies generally support synergistic effects between NAD^+^ precursors and exercise, human trials have produced mixed outcomes, with improvements more evident in individuals with impaired metabolic or redox homeostasis than in healthy populations. Future studies should determine optimal combinations of NAD^+^ precursor type, dosage, exercise modality, and intervention duration to maximize functional benefits during aging.

Collectively, current evidence suggests that NAD^+^ precursors may influence some metabolic adaptations induced by exercise, particularly in aged organisms with impaired NAD^+^ homeostasis. However, additive effects are not consistently demonstrated in humans, and the optimal combination of precursor type, dose, exercise modality, and intervention duration remains unresolved.

### 9.2 Critical appraisal of current evidence on vitamin B3–derived NAD^+^ precursors supplementation with exercise in aging

The current evidence regarding the combined effects of NAD^+^ precursor supplementation and exercise in aging remains promising but insufficient for definitive conclusions. Most available studies are characterized by small sample sizes, short intervention periods, and heterogeneous experimental designs, limiting the ability to determine whether NAD^+^ augmentation provides clinically meaningful benefits beyond those achieved by exercise alone. Human trials have generally involved limited cohorts and short-term interventions, whereas animal studies provide stronger mechanistic evidence but have restricted translational relevance.

A major limitation is the variability among interventions, including differences in NAD^+^ precursor type (NR, NMN, NAM, or niacin), dosage, treatment duration, and exercise protocols. Exercise-induced adaptations are strongly influenced by modality, intensity, duration, and baseline physical capacity; therefore, inconsistent exercise paradigms make direct comparison among studies challenging. In addition, most investigations have relied on molecular or metabolic biomarkers, including NAD^+^ concentrations, NAD^+^/NADH ratio, NAMPT expression, and oxidative stress markers, rather than clinically relevant outcomes such as long-term preservation of mobility, independence, or prevention of age-related functional decline. Importantly, evidence from skeletal muscle should not be directly extrapolated to the brain. Although skeletal muscle and neurons are both highly metabolically active tissues, they differ substantially in cellular composition, metabolic regulation, antioxidant capacity, and vulnerability to age-related stress. Therefore, improvements in muscle NAD^+^ metabolism or physical performance cannot automatically predict similar effects on neuronal resilience or cognitive aging. Another unresolved issue concerns whether NAD^+^ supplementation enhances normal exercise adaptation or primarily compensates for age-associated metabolic impairment. Some studies suggest additive or synergistic effects between NAD^+^ precursors and exercise, whereas others demonstrate limited additional benefits compared with exercise alone. These discrepancies may reflect differences in baseline NAD^+^ availability, biological age, mitochondrial status, and the capacity of tissues to respond to metabolic interventions. Future studies should prioritize larger randomized controlled trials with standardized exercise interventions, longer follow-up periods, and clinically meaningful functional outcomes. Particular attention should be given to identifying responder populations, optimal dosing strategies, and whether combined NAD^+^ and exercise interventions can prevent rather than merely attenuate age-related decline. Furthermore, the interaction between NAD^+^ precursor supplementation and exercise appears to be highly dependent on exercise modality, training status, age, and baseline metabolic condition. Although exercise independently activates endogenous NAD^+^ biosynthesis and mitochondrial adaptation pathways, current evidence is insufficient to determine whether exogenous NAD^+^ precursors provide additional benefits beyond exercise-induced adaptations. Future studies should therefore incorporate standardized exercise protocols and stratify participants according to metabolic status and physical capacity to determine whether combined interventions produce clinically meaningful advantages (106–108).

### 9.3 Molecular mechanisms of NAD^+^ repletion with exercise in aging

The interaction between NAD^+^ precursor supplementation and exercise may influence metabolic resilience during aging through convergent effects on cellular energy regulation, mitochondrial adaptation, and stress-response pathways. Exercise increases energetic demand and activates metabolic sensors, whereas NAD^+^ precursors provide additional substrate availability for NAD^+^-dependent enzymes. Together, these interventions may promote adaptive responses in aging tissues characterized by impaired metabolic flexibility. A central mechanism involves restoration of NAD^+^ availability and activation of NAD^+^-dependent regulators, particularly SIRT1 and SIRT3. These sirtuins contribute to mitochondrial adaptation by regulating oxidative metabolism, mitochondrial biogenesis, and cellular stress resistance. Importantly, NAD^+^ does not directly induce improvements in muscle performance or neuronal function; rather, any functional effects are likely to occur through downstream processes involving mitochondrial remodeling, redox regulation, and metabolic adaptation.

Exercise-induced activation of AMPK may further interact with NAD^+^ metabolism by enhancing NAMPT-dependent NAD^+^ salvage. The AMPK–NAMPT–NAD^+^–sirtuin axis may contribute to improved mitochondrial efficiency, glucose utilization, and fatty acid oxidation, thereby supporting energy homeostasis in aging tissues. In addition, exercise-induced oxygen-sensing mechanisms, including HIF-1α signaling, may contribute to metabolic adaptation and vascular responses, with emerging evidence suggesting functional interactions between HIF-1α activity and NAD^+^ metabolism. At the skeletal muscle level, NAD^+^ restoration has been associated with changes in mitochondrial respiration, energy availability, and resistance to metabolic stress. Increased NAD^+^ availability may also support antioxidant defense systems by maintaining redox balance and modulating stress-responsive pathways, including nuclear factor erythroid 2–related factor 2 (Nrf2)-dependent antioxidant responses. However, these molecular adaptations should not be interpreted as universally equivalent to functional improvement, as responses depend on biological age, baseline NAD^+^ status, exercise characteristics, and tissue-specific metabolic demands. Furthermore, although skeletal muscle represents an important model for understanding systemic metabolic adaptation, its responses cannot be directly extrapolated to neuronal tissues.

Recent results further highlight the necessity of interpreting NAD^+^ biology in a tissue-specific manner. While NAD^+^ depletion is commonly seen with aging, diminished NAD^+^ availability in skeletal muscle does not necessarily trigger a faster decline in function or worsen muscle performance, indicating the involvement of compensatory mechanisms and responses that vary by tissue. Consequently, beneficial changes in skeletal muscle metabolism that follow NAD^+^ precursor supplementation and exercise should not be taken as evidence of equivalent effects in the central nervous system. Neuronal tissues have distinct energetic demands, limited regenerative ability, and heightened susceptibility to mitochondrial dysfunction and oxidative stress, which can produce different outcomes in the context of NAD^+^ imbalance (18).

The brain exhibits distinct metabolic requirements, cellular composition, and vulnerability to oxidative stress, requiring specific investigation of whether exercise-induced NAD^+^ restoration is associated with preservation of neuronal resilience and cognitive function. Overall, combined NAD^+^ precursor supplementation and exercise may represent a biologically plausible complementary approach for influencing metabolic flexibility and resilience during aging. Current evidence supports a potential complementary interaction between these interventions rather than a universally established synergistic effect. Future studies should determine whether optimized NAD^+^-exercise protocols can delay age-related functional decline, improve tissue-specific adaptation, and reduce vulnerability to neurodegenerative processes. A key unresolved issue is whether NAD^+^-dependent metabolic adaptation can affect structural factors that support cognitive resilience, especially dendritic spine maintenance. Dendritic spines are particularly vulnerable to oxidative stress due to their tight coupling with mitochondria and specialized calcium-regulating components such as the spine apparatus. Because spine loss is a common hallmark of aging and NDDs, future studies should test whether NAD^+^ replenishment and exercise-driven antioxidant responses are associated with preservation of synaptic structure and cognitive performance. Combining NAD^+^ biomarkers with neuroimaging, electrophysiological evaluations, and measures of synaptic integrity may yield important insights into how metabolic regulation relates to brain health (19–21).

## 10 Clinical evidence and translational perspectives

Clinical investigations evaluating NAD^+^-enhancing interventions, particularly vitamin B3-derived precursors including NR, NMN, and niacin, have expanded substantially in recent years. Human studies generally indicate favorable short-term safety profiles across different dosing regimens, with adverse effects typically limited to mild gastrointestinal symptoms or flushing in the case of niacin. Importantly, clinical trials consistently demonstrate that NR and NMN supplementation can increase circulating NAD^+^ metabolites; however, whether peripheral NAD^+^ augmentation translates into sufficient and sustained NAD^+^ restoration within the central nervous system, and ultimately into clinically meaningful neurological benefits, remains unresolved. Emerging evidence suggests that exercise represents an important physiological regulator of NAD^+^ metabolism in humans. Acute and chronic physical activity have been associated with improved mitochondrial function, enhanced metabolic flexibility, increased sirtuin activity, and favorable modulation of inflammatory and oxidative stress pathways. However, direct clinical evidence demonstrating additive or synergistic effects between NAD^+^ precursors and structured exercise interventions remains limited. Thus, exercise should not currently be considered merely as a means of enhancing NAD^+^ levels, but rather as a multifactorial intervention whose effects involve mitochondrial, vascular, neurotrophic, inflammatory, and metabolic adaptations. Optimal exercise parameters, including intensity, duration, and modality, as well as the characteristics of individuals most likely to benefit from combined interventions, have not yet been established.

From a translational perspective, reliable biomarkers are essential for monitoring biological responses to NAD^+^-targeted interventions. Current approaches include measurements of circulating NAD^+^ metabolites, NAD^+^/NADH ratios, inflammatory mediators, mitochondrial-related markers, and multi-omics approaches such as metabolomics and transcriptomics. Nevertheless, considerable variability exists among analytical methods, biological samples, and normalization strategies, limiting cross-study comparisons and clinical interpretation. In particular, circulating NAD^+^ concentrations should not be regarded as direct surrogates of brain NAD^+^ status or neuronal target engagement. Future studies should therefore incorporate brain-relevant biomarkers, including neuroimaging, neuronal extracellular vesicles, and validated measures of synaptic, metabolic, and neuroinflammatory changes. Despite promising metabolic and molecular findings, current clinical evidence remains preliminary due to small sample sizes, short intervention periods, heterogeneous populations, differences in precursor type and dosing, and inconsistent outcome measures. Moreover, evidence directly supporting cognitive improvement, prevention of neurodegeneration, or disease modification in AD and PD remains insufficient. Accordingly, the current evidence supports NAD^+^-enhancing interventions primarily as biologically active and potentially beneficial metabolic strategies rather than established disease-modifying therapies. Future large-scale randomized controlled trials incorporating standardized biomarkers, long-term follow-up, neuroimaging approaches, and clinically relevant cognitive and functional outcomes are required to determine whether NAD^+^-enhancing strategies can provide meaningful benefits for brain aging and the prevention or modification of NDDs.

## 11 Therapeutic challenges and limitations

Despite substantial advances in understanding NAD^+^ biology and developing vitamin B3-derived strategies, several challenges continue to limit their clinical translation for brain aging and NDDs. A major unresolved issue concerns bioavailability and tissue-specific delivery. Although NR and NMN can effectively increase systemic NAD^+^ metabolites, the extent to which these interventions produce sustained increases in neuronal NAD^+^ availability remains insufficiently characterized in humans. Therefore, the magnitude of potential central nervous system benefits and their relevance to cognitive outcomes remains uncertain. Dose optimization represents another important challenge. Current studies employ heterogeneous supplementation protocols with considerable variation in precursor type, dose, formulation, intervention duration, and participant characteristics, making it difficult to define optimal therapeutic ranges for aging populations or individuals with neurodegenerative conditions. Future trials should incorporate standardized dosing strategies and evaluate whether NAD^+^ precursors exert differential effects depending on disease stage, baseline NAD^+^ status, metabolic condition, and concurrent lifestyle interventions such as exercise.

Clinical heterogeneity further complicates interpretation of current findings. Differences in age, baseline metabolic health, genetic background, disease severity, lifestyle factors, and underlying biological mechanisms may substantially influence individual responses to NAD^+^ augmentation. This variability highlights the need for precision medicine approaches incorporating patient stratification and predictive biomarkers to identify individuals most likely to benefit. Long-term safety remains an additional consideration, particularly for chronic supplementation in older populations. Although short-term clinical studies generally demonstrate good tolerability, the consequences of sustained NAD^+^ elevation on cellular signaling networks, metabolic regulation, and other physiological processes require evaluation through longer-term monitoring. Current evidence does not indicate major safety concerns; however, the absence of observed adverse effects in relatively short clinical studies does not exclude potential long-term consequences. Finally, host–microbiome interactions represent an emerging factor that may influence the metabolism and biological effects of NAD^+^ precursors. Differences in microbial composition may affect nutrient metabolism, inflammatory status, and responses to lifestyle interventions, including exercise. However, whether microbiome alterations represent a determinant of therapeutic responsiveness or a secondary consequence of improved metabolic status remains unclear and requires validation in well-controlled human studies. Overall, overcoming these challenges will require large-scale longitudinal trials integrating standardized NAD^+^ measurements, brain-relevant biomarkers, neuroimaging, multi-omics approaches, and clinically meaningful cognitive outcomes to determine whether NAD^+^-targeted interventions can provide durable benefits for brain aging and NDD prevention or whether their primary effects remain limited to modulation of intermediate metabolic and molecular pathways.

## 12 Future directions

Future research on NAD^+^ biology and brain aging should move beyond demonstrating biological effects toward establishing clinically meaningful, evidence-based, and individualized therapeutic strategies. A major priority will be the development of precision medicine approaches capable of identifying individuals who are most likely to benefit from NAD^+^-enhancing interventions. Patient stratification based on age, genetic background, metabolic status, baseline NAD^+^ availability, disease stage, and molecular profiles may improve therapeutic targeting and help explain the heterogeneous responses observed in current clinical studies. An important emerging area involves understanding the interaction between NAD^+^ metabolism and the gut–brain axis. Although preclinical evidence suggests that microbiome composition may influence NAD^+^ metabolism, inflammation, and neuroactive metabolite production, the clinical relevance of microbiome-directed NAD^+^ interventions remains largely hypothetical and requires validation in well-controlled human studies. Future studies should clarify whether dietary modulation, targeted microbial interventions, or other microbiome-based strategies can enhance the efficacy and consistency of NAD^+^-related therapies in humans.

Combination approaches represent another promising direction. Given that exercise independently stimulates NAD^+^ metabolism, mitochondrial adaptation, and neuroplasticity-related pathways, integrating NAD^+^ precursors with structured exercise programs may provide complementary benefits. However, current evidence is insufficient to confirm additive or synergistic effects, and well-designed clinical trials are required to determine whether such combinations provide advantages beyond exercise or supplementation alone and to define optimal exercise characteristics, including intensity, duration, and modality. Future clinical development will also depend on the establishment of validated biomarkers and standardized assessment frameworks. Integrating measurements of NAD^+^ metabolites, mitochondrial function, inflammatory signatures, neuroimaging markers, cognitive outcomes, and multi-omics profiles may enable more accurate monitoring of biological responses and therapeutic efficacy. Importantly, peripheral NAD^+^ measurements should not be assumed to directly represent brain NAD^+^ status; therefore, future studies should prioritize brain-relevant biomarkers and functional neurological outcomes.

A further unresolved issue is whether NAD^+^-focused interventions can directly affect synaptic structural resilience during aging and neurodegeneration. While mitochondrial dysfunction, oxidative stress, and disrupted metabolic homeostasis are well recognized as drivers of cognitive decline, the connection between NAD^+^ restoration and the preservation of dendritic spines is still not well characterized. Dendritic spines are key determinants of neuronal connectivity and plasticity, and they are especially susceptible to oxidative stress because of their close relationship with mitochondria and specialized calcium-regulating structures, including the spine apparatus. Future work should test whether NAD^+^ precursor supplementation, exercise-linked metabolic adaptation, or a combination of both can maintain dendritic spine density, calcium homeostasis, and synaptic performance. Combining NAD^+^-related biomarkers with advanced neuroimaging, electrophysiological methods, and direct evaluations of synaptic architecture may clarify whether metabolic restoration can yield substantive cognitive benefits. Importantly, long-term randomized controlled studies with adequately powered cohorts are required to determine whether NAD^+^ augmentation can modify disease progression rather than merely improve intermediate molecular markers. Ultimately, the future of NAD^+^-based interventions in neuroaging will likely rely on multimodal and personalized strategies that integrate metabolic regulation, lifestyle modification, and molecular biomarkers. Such approaches may provide a more realistic framework for promoting cognitive resilience and extending brain healthspan while addressing the biological complexity of aging and NDDs. Several unresolved questions remain, including the optimal dose and duration of NAD^+^ precursor supplementation, the long-term safety of sustained NAD^+^ elevation, the development of validated biomarkers reflecting brain NAD^+^ status, and the identification of patient populations most likely to benefit from NAD^+^-targeted interventions. Future clinical trials should also prioritize multimodal designs integrating NAD^+^ precursors with structured exercise programs and microbiome-directed interventions to determine whether combined approaches provide clinically meaningful benefits compared with single interventions.

## 13 Conclusion

Available evidence supports the concept that declining NAD^+^ availability represents an important metabolic feature associated with brain aging and NDDs. Vitamin B3-derived NAD^+^ precursors, including NAM, NR, nicotinic acid, and NMN, have demonstrated the ability to increase NAD^+^ availability and regulate multiple cellular processes involved in neuronal maintenance, including mitochondrial function, DNA repair, autophagy, and redox homeostasis. However, the biological effects of NAD^+^ restoration should be interpreted primarily as modulation of downstream NAD^+^-dependent signaling networks rather than as direct enhancement of neuronal function. Preclinical studies in models of AD, PD, and age-associated cognitive impairment suggest that NAD^+^ restoration may reduce pathological processes such as neuroinflammation, oxidative stress, impaired mitochondrial function, and synaptic dysfunction. Nevertheless, most supporting evidence remains derived from cellular and animal models, and the extent to which these molecular adaptations translate into clinically meaningful cognitive preservation or disease modification in humans remains uncertain.

Physical exercise represents an important physiological modulator that interacts with NAD^+^ metabolism through mechanisms involving mitochondrial adaptation, sirtuin activation, vascular regulation, and neurotrophic signaling. Although exercise may enhance endogenous NAD^+^ metabolism and complement NAD^+^ precursor interventions, current clinical evidence is insufficient to establish consistent synergistic effects between exercise and NAD^+^ supplementation. Similarly, emerging findings indicate that gut microbiome composition may influence NAD^+^ metabolism and host responses; however, the causal contribution of microbiome-mediated mechanisms to NAD^+^-related neuroprotection remains largely unresolved and requires validation in well-controlled human studies. Overall, NAD^+^-targeted interventions represent a promising but still developing strategy for promoting brain resilience during aging. Current evidence supports NAD^+^ precursors as metabolic modulators with potential to improve cellular resilience; however, they should not yet be considered established preventive or therapeutic agents for NDDs. Future progress will depend on adequately powered longitudinal clinical trials incorporating standardized biomarkers, neuroimaging approaches, cognitive outcomes, and patient stratification strategies. Future studies should also address unresolved questions regarding optimal precursor selection, dosing, treatment duration, brain-specific NAD^+^ availability, and long-term safety of sustained NAD^+^ enhancement. A personalized framework integrating NAD^+^ modulation, lifestyle interventions, and biological profiling may ultimately provide a more effective approach for preserving cognitive health and reducing vulnerability to NDDs. Such precision-based strategies may help identify individuals most likely to benefit while avoiding the assumption that NAD^+^ restoration produces uniform effects across different aging populations, tissues, or disease stages.
